# Human Brain Cell‐Type‐Specific Aging Clocks Based on Single‐Nuclei Transcriptomics

**DOI:** 10.1002/advs.202506109

**Published:** 2025-08-29

**Authors:** Chandramouli Muralidharan, Enikő Zakar‐Polyák, Anita Adami, Anna A. Abbas, Yogita Sharma, Raquel Garza, Jenny G. Johansson, Diahann A. M. Atacho, Éva Renner, Miklós Palkovits, Csaba Kerepesi, Johan Jakobsson, Karolina Pircs

**Affiliations:** ^1^ Laboratory of Molecular Neurogenetics Department of Experimental Medical Science Wallenberg Neuroscience Center and Lund Stem Cell Center Lund University Lund 221 84 Sweden; ^2^ Institute of Translational Medicine Semmelweis University Budapest 1094 Hungary; ^3^ Hungarian Centre of Excellence for Molecular Medicine – Semmelweis University (HCEMM‐SU) Neurobiology and Neurodegenerative Diseases Research Group Budapest 1094 Hungary; ^4^ Institute for Computer Science and Control (SZTAKI) Hungarian Research Network (HUN‐REN) Budapest 1111 Hungary; ^5^ Doctoral School of Informatics Eötvös Loránd University Budapest 1053 Hungary; ^6^ HUN‐REN‐SZTAKI‐SU Rejuvenation Research Group Office for Supported Research Groups (TKI) Hungarian Research Network (HUN‐REN) Budapest 1052 Hungary; ^7^ Human Brain Tissue Bank Semmelweis University Budapest 1094 Hungary

**Keywords:** aging clocks, biological clocks, human brain aging, single nuclei sequencing, transcriptomic clocks

## Abstract

Aging is the primary risk factor for most neurodegenerative diseases, yet the cell‐type‐specific progression of brain aging remains poorly understood. Here, human cell‐type‐specific transcriptomic aging clocks are developed using high‐quality single‐nucleus RNA sequencing data from post mortem human prefrontal cortex tissue of 31 donors aged 18–94 years, encompassing 73,941 high‐quality nuclei. Distinct transcriptomic changes are observed across major cell types, including upregulation of inflammatory response genes in microglia from older samples. Aging clocks trained on each major cell type accurately predict chronological age, capture biologically relevant pathways, and remain robust in independent single‐nucleus RNA‐sequencing datasets, underscoring their broad applicability. Notably, cell‐type‐specific age acceleration is identified in individuals with Alzheimer's disease and schizophrenia, suggesting altered aging trajectories in these conditions. These findings demonstrate the feasibility of cell‐type‐specific transcriptomic clocks to measure biological aging in the human brain and highlight potential mechanisms of selective vulnerability in neurodegenerative diseases.

## Graphical abstract

Muralidharan and colleagues develop cell‐type‐specific transcriptomic aging clocks using single‐nucleus RNA sequencing of human post mortem prefrontal cortex samples. These clocks accurately predict age and identify distinct aging trajectories in specific brain cell types. Their method also detects accelerated aging in Alzheimer's disease and schizophrenia, offering insight into differential brain aging across health and disease.

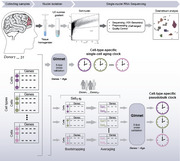

## 1 Introduction

Aging involves complex molecular changes that gradually lead to the functional decline of cells and organs, including the different cell types in the brain.^[^ ^1^, ^2^ ^]^ Aging is a major risk factor for most neurodegenerative diseases, including Alzheimer's and Parkinson's disease.^[^ ^3^ ^]^ However, our understanding of how different cell types age in the brain, the molecular mechanisms underlying their aging processes and how these changes contribute to the disease pathophysiology remains limited. To better understand these processes and how they are altered in disease, it will be important to develop tools that accurately measure molecular age‐related changes in the different cell types in the brain.

In recent years, several molecular aging clocks have been developed that use different types of omics data in combination with machine learning algorithms to predict biological age.^[^ ^4^, ^5^, ^6^, ^7^, ^8^, ^9^ ^]^ These tools offer a quantitative measure of aging beyond chronological age, enabling the identification of individuals or tissues at risk for accelerated aging and providing insights into underlying molecular pathways. With few exceptions,^[^ ^4^, ^10^, ^11^, ^12^, ^13^ ^]^ these aging clocks are derived from bulk tissue^[^ ^6^, ^7^, ^8^, ^14^, ^15^ ^]^ and therefore lack cell‐type‐specific resolution. With an increasing number of studies pointing to cell‐type‐specific mechanisms of aging in the human brain,^[^ ^16^, ^17^, ^18^, ^19^, ^20^ ^]^ such bulk‐trained clocks have a major limitation in accurately capturing age‐related changes. In a recent study in the mouse brain, cell‐type‐specific transcriptomic aging clocks based on single‐cell RNA sequencing were developed. They not only accurately predicted the chronological age for different cell types but also modelled the reversal of aging with exercise and rejuvenation.^[^ ^4^ ^]^ This highlights the need to develop human brain cell‐type‐specific aging clocks that can predict the age of individual cells from different cell types. Further, such a tool would have the potential to identify the selective vulnerability of specific cell types in human age‐related neurodegenerative disorders.

In this study, we performed single‐nuclei RNA sequencing (snRNA‐seq) on 31 post mortem human prefrontal cortex tissues from young, middle‐aged, and old donors. Using this dataset, we found cell‐type‐specific age‐related transcriptomic differences, allowing us to develop human single‐nuclei‐based transcriptomic aging clocks for different cell types in the brain, which could be validated using publicly available snRNA‐seq datasets. In addition, our approach showed cell‐type‐specific age acceleration in Alzheimer's disease and schizophrenia, highlighting its utility in uncovering selective cellular vulnerability in age‐related neurodegenerative disorders.

## 2 Results

### 2.1 Single‐Nuclei RNA Sequencing of a Human Aging Cohort

**Figure 1:**
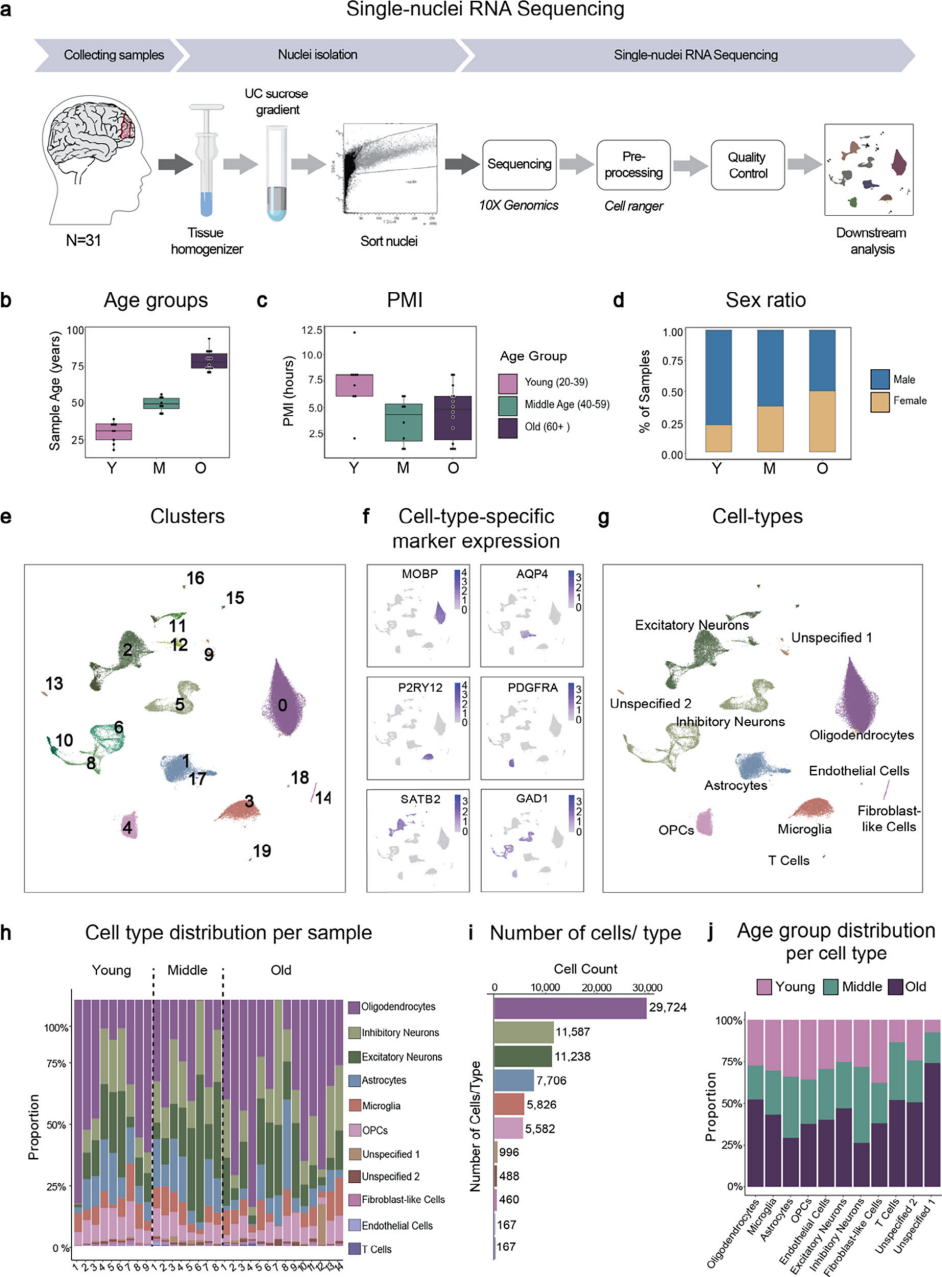
Single‐nucleus RNA sequencing of human post mortem prefrontal cortex tissue from an aging cohort. a) Schematic of the experimental workflow for snRNA‐seq. Nuclei were isolated and sorted from frozen human post mortem prefrontal cortex tissue samples from 31 donors, followed by snRNA‐seq and downstream analysis. b) Box plot showing the age distribution of samples in the different age groups. Samples in the young age group (Y, *n* = 8), range 18–39 years, 40–59 years in the middle‐aged group (M, *n* = 7), and 60–94 years in the old age group (O, *n* = 14). c) Box plot showing the distribution of the post mortem interval (PMI) of samples in the different age groups. d) Bar chart showing the proportion of the samples of each sex in the different age groups. e) UMAP plot annotated with the 19 identified cell clusters. f) Projection of gene expression of canonical markers of different cell types in the adult human prefrontal cortex. g) UMAP plot annotated with the identified cell types. h) Bar plot showing the proportion of cells of different cell types in all the samples. i) Bar graph showing the number of cells in the dataset from each identified cell type. j) Bar graph showing the proportion of cells of different age groups within each cell type.

To study cell‐type‐specific changes during human brain aging, we performed snRNA‐seq on post mortem human prefrontal cortex tissue derived from 31 donors aged between 18 and 94 years at death (**Figure** **1**a–d, **Table** **1**; Data S1a, Supporting Information). We sequenced fresh frozen punch biopsies originating from the ventrolateral prefrontal cortex or the middle frontal gyrus, two brain regions that are a part of the prefrontal cortex, which has been implicated in age‐related neurodegenerative disorders such as Alzheimer's disease.^[^ ^21^, ^22^, ^23^, ^24^ ^]^ Tissue samples were distributed across three age groups, including tissue coming from young (18–39 years), middle‐aged (40–59 years), and old (60 + years) individuals (Figure 1b). Tissue was collected with a short median post mortem interval (PMI) of 4.5 (2–12) h (Figure 1c) and was obtained from both sexes (Figure 1d). After sequencing and quality control, we obtained data from 73,941 high‐quality nuclei (Figure S1a–d and Data S1b, Supporting Information). We performed an unbiased clustering resulting in 19 distinct cell clusters (Figure 1e). Using the expression of cell‐type‐specific marker genes, we identified all the major cell types present in the human prefrontal cortex, namely, oligodendrocytes, astrocytes, oligodendrocyte progenitor cells (OPCs), microglia, inhibitory neurons, and excitatory neurons (Figure 1f,g; Figure S1e and Data S2, Supporting Information). All the major cell types were represented across all the samples (Figure 1h; Figure S1f and Data S1c, Supporting Information) and across the different age groups (Figure 1j). In addition, these cell types were the most abundant in the dataset (Figure 1i), making them suitable for comparison.

**Table 1:** Demographic and clinical data of the human prefrontal cortical samples.

Sample ID	Age [years]	Sex	PMI [h]	Brain Region	Cause of Death
y1	22	Male	8	Middle frontal gyrus	suicide
y2	36	Female	2	Middle frontal gyrus	suicide
y3	31	Male	8	Middle frontal gyrus	suicide
y4	31	Male	7	Middle frontal gyrus	suicide
y5	36	Male	6	Ventrolateral prefrontal cortex	suicide
y6	35	Male	6	Middle frontal gyrus	suicide
y7	18	Male	8	Middle frontal gyrus	suicide
y8	25	Male	8	Middle frontal gyrus	suicide
y9	39	Female	12	Middle frontal gyrus	suicide (hanging)
m1	42	Male	3.5	Ventrolateral prefrontal cortex	cardiac insufficiency
m2	55	Male	1	Ventrolateral prefrontal cortex	acute myocardial infarction
m3	47	Male	1	Ventrolateral prefrontal cortex	cardiac insufficiency
m4	50	Male	2	Ventrolateral prefrontal cortex	myocardial infarction
m5	53	Male	5	Ventrolateral prefrontal cortex	pulmonary embolism
m6	56	Female	6	Ventrolateral prefrontal cortex	cardiorespiratory insufficiency
m7	49	Female	6	Ventrolateral prefrontal cortex	suicide (drug overdose)
m8	44	Female	5	Dorsolateral prefrontal cortex	acute cardiac insufficiency
o1	86	Male	3	Ventrolateral prefrontal cortex	acute cardiac insufficiency
o2	73	Male	6	Ventrolateral prefrontal cortex	acute cardiac failure; general arteriosclerosis
o3	81	Male	5	Ventrolateral prefrontal cortex	acute ischaemic heart failure; cardiogenic shock
o4	80	Male	4.5	Ventrolateral prefrontal cortex	cardiorespiratory insufficiency
o5	76	Male	5.5	Ventrolateral prefrontal cortex	stroke^a)^
o6	94	Female	6	Ventrolateral prefrontal cortex	cardiac failure
o7	74	Female	1.5	Ventrolateral prefrontal cortex	cardiac insufficiency; pulmonary embolism
o8	70	Female	7	Ventrolateral prefrontal cortex	stroke^a)^
o9	75	Male	8	Ventrolateral prefrontal cortex	stroke^a)^
o10	74	Male	1	Ventrolateral prefrontal cortex	acute cardiac insufficiency
o11	80	Female	1	Ventrolateral prefrontal cortex	acute respiratory insufficiency
o12	85	Female	4	Ventrolateral prefrontal cortex	acute cardiorespiratory; renal insufficiency
o13	72	Female	1	Ventrolateral prefrontal cortex	pulmonary embolism
o14	85	Female	8	Ventrolateral prefrontal cortex	stroke^a)^; hypertension

^a)^ The microdissected region was distinct from the stroke‐affected region.

### 2.2 Age‐Related Transcriptomic Changes in Microglia and Astrocytes

**Figure 2:**
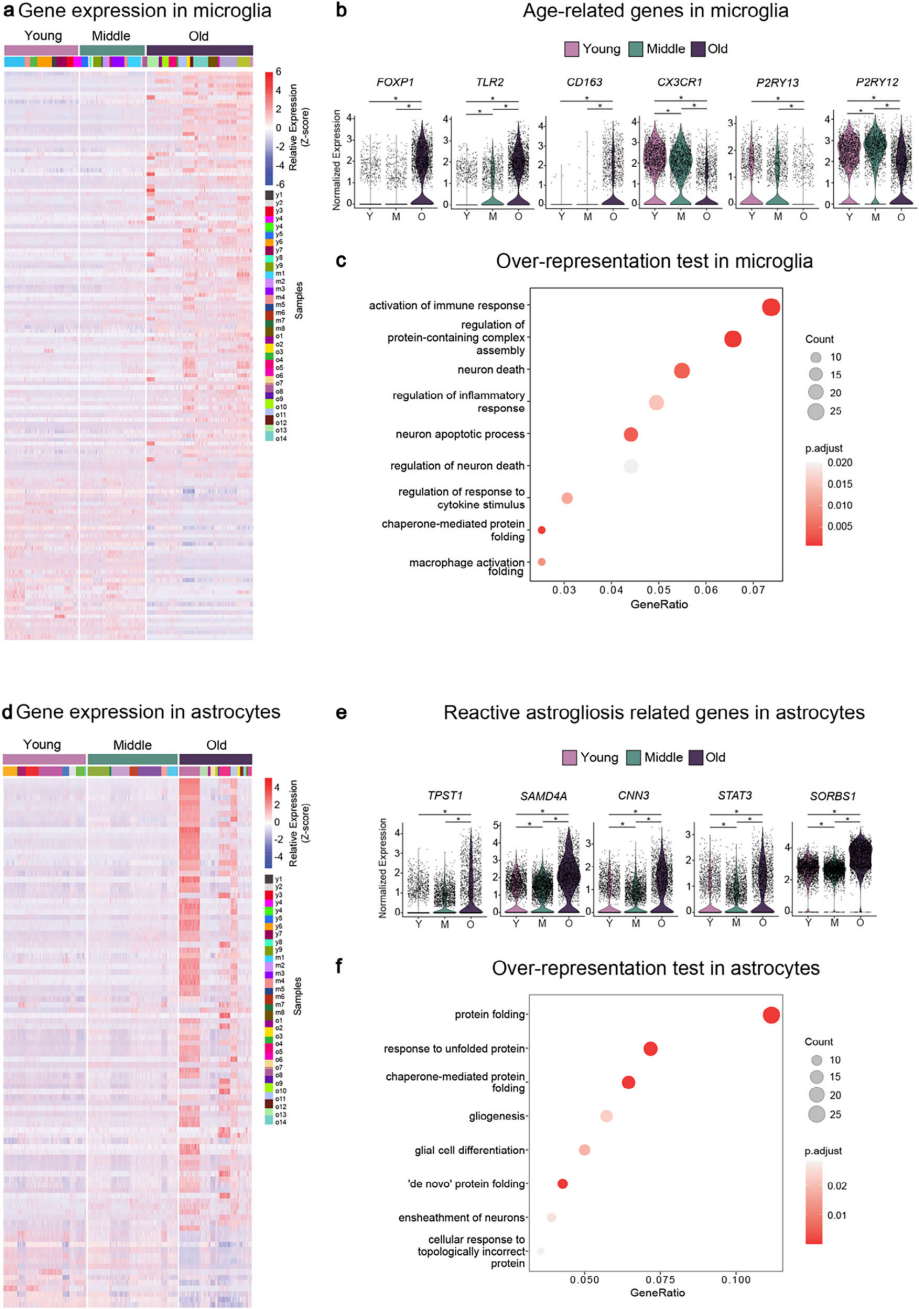
Age‐related transcriptomic changes are present in microglia and astrocytes. a) Heatmap showing the expression of the top differentially expressed genes (−0.75 > average log2 fold‐change > 0.75, adj. *p*‐value < 0.05) in microglia when comparing old versus young age groups. Different colours below the young, middle, and old ages bar represent cells originating from different individuals. b) Violin plots showing the expression of several previously identified microglial aging genes. c) Plot showing selected gene ontology terms enriched from genes differentially expressed in old microglia. d) Heatmap showing the expression of the top differentially expressed genes (−0.75 > average log2 fold‐change > 0.75, adj. *p*‐value < 0.05) in astrocytes when comparing old versus young age groups. Different colours below the young, middle, and old ages bar represent cells originating from different individuals. e) Violin plots showing the expression of selected reactive astrogliosis genes. f) Plots showing selected gene ontology terms enriched from genes differentially expressed in old astrocytes. ^*^ *P* < 0.05; Wilcoxon rank sum test was used for each comparison in b and e, while ClusterProfiler's gene‐overrepresentation analysis, along with Benjamini‐Hochberg correction for multiplicity, was used in e and f.

First, we investigated cell‐type‐specific age‐related changes in our dataset by performing differential gene expression analysis between the different age groups. We found distinct age‐related transcriptomic changes in each of the different cell types (**Figure** **2**a,d; Figure S2a,c,d,f, and Data S3, Supporting Information). For example, in microglia, we observed inflammatory response genes significantly upregulated in the old age group when compared to the young or the middle age groups, such as *FOXP1*, *TLR2*, and *CD163*, while homeostatic microglial markers, such as *CX3CR1*, *P2RY12*, and *P2RY13*, were downregulated (Figure 2b; Data S3g–l, Supporting Information). Gene over‐representation test of the differentially expressed genes revealed an enrichment of terms related to inflammatory response (Figure 2c; Figure S2a and Data S4d–f, Supporting Information). These observations are consistent with previous studies on microglial aging, which reported an increased inflammatory response during aging.^[^ ^25^, ^26^ ^]^ In astrocytes, we found an upregulation of genes associated with reactive astrogliosis, such as *TPST1*, *SAMD4A*, *CNN3*, *STAT3*, and *SORBS1* (Figure 2e; Data S3m–r, Supporting Information), in the old group when compared to the young or middle‐aged groups. This was in line with previous studies showing that reactive astrogliosis occurs during inflammation in the aging brain.^[^ ^27^, ^28^, ^29^ ^]^ Gene ontology (GO) analysis on differentially expressed genes in each of the major cell types revealed enrichment of terms related to protein misfolding, a hallmark of brain aging,^[^ ^1^, ^2^ ^]^ were enriched in astrocytes (Figure 2f; Data S4g,h, Supporting Information). In oligodendrocytes, the chaperone‐mediated protein‐folding term was enriched (Figure S2b and Data S4a–c, Supporting Information). On the other hand, in excitatory neurons terms related to protein translation were enriched (Figure S2e and Data S4j–l, Supporting Information), while in inhibitory neurons and OPCs, no significant terms were enriched in old versus young age group comparisons.

In summary, the results demonstrated that age‐related transcriptomic changes are present and can be detected in our snRNA‐seq dataset. Importantly, the transcriptional response to aging is different in each cell type, highlighting the relevance of the development of cell‐type‐specific transcriptional clock algorithms using this dataset.

### 2.3 Development and Evaluation of Cell‐Type‐Specific Transcriptomic Aging Clocks for the Human Brain

**Figure 3:**
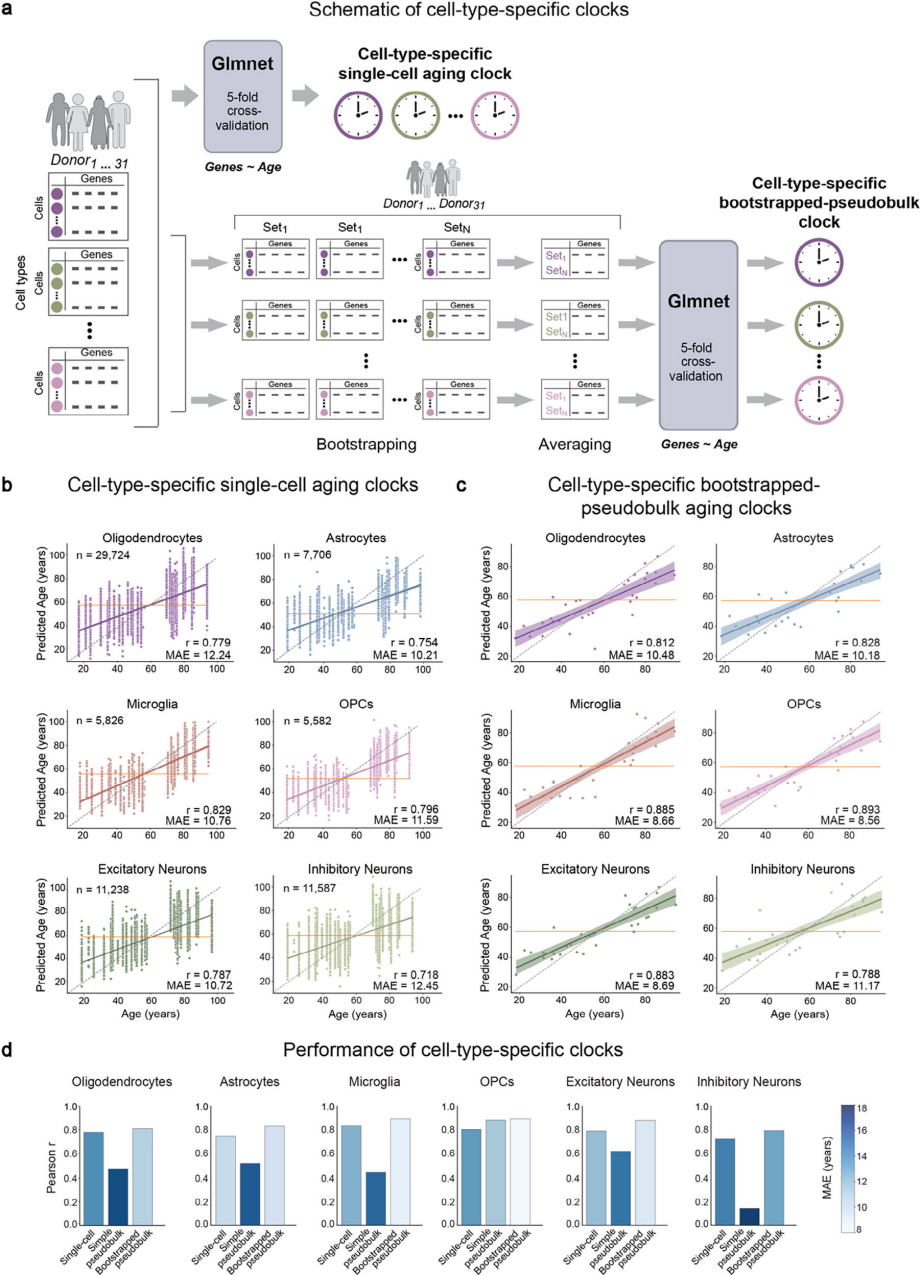
Development and evaluation of cell‐type‐specific transcriptomic aging clocks for the human brain. a) Schematic representation of the different approaches in the development of the aging clocks using the ElasticNet regression model via the GlmNet algorithm. Cell‐type‐specific single‐cell aging clocks were trained and tested directly on the (log‐normalised) single‐cell gene expression values, while the cell‐type‐specific (simple or bootstrapped‐) pseudobulk aging clocks were trained and tested on aggregated gene expression data of cells derived from a given cell type (b, c) Relationship between chronological age and the age predicted from the test rounds of (b) cell‐type‐specific single‐cell aging clocks and (c) cell‐type‐specific bootstrapped‐pseudobulk clocks. The horizontal line (orange) represents predictions from a naïve mean prediction model. d) Bar plots showing Pearson's correlation coefficients and mean absolute errors (MAE, represented by the intensity of blue colour in the bars) of each approach in each cell type. All correlation tests were performed using the stats.pearsonr function in SciPy, with significance based on a *p*‐value < 0.05.

To measure the extent of aging in each cell type based on transcriptomic information, we trained ElasticNet regression models^[^ ^30^, ^31^ ^]^ on our dataset via fivefold cross‐validation using genes as features and age as the target. We focused on the most abundant cell types in the dataset, namely, oligodendrocytes, microglia, astrocytes, OPCs, inhibitory neurons, and excitatory neurons. We used three different approaches to train the aging clock models for each cell type. In the first approach, we used the log‐normalized gene expression values of individual cells, while in the second approach, we used the average of the log‐normalized gene expression across all cells from a given donor and cell type, resulting in cell‐type‐specific simple pseudobulk samples (**Figure** **3**a; Data S5a–l, Supporting Information). Given that the averaging in the simple pseudobulk approach would result in only one data point per donor per cell type, thereby reducing variance, we further used a third approach similar to what was previously described in Buckley et al.^[^ ^4^ ^]^ We randomly sampled (i.e., bootstrapped) a fixed number of cells and averaged the expressions into cell‐type‐specific bootstrapped‐pseudobulk samples (Figure 3a), resulting in multiple data points per donor per cell type (Data S5m–r, Supporting Information).

We found that the cell‐type‐specific single‐cell aging clocks showed a statistically significant positive correlation between the predicted age and the chronological age for all cell types examined, with correlation coefficients 0.74–0.83 and mean absolute errors (MAE) of 10.2–12.2 years (Figure 3b; Data S6a–f and S7, Supporting Information). The age prediction performance was improved when we tested the cell‐type‐specific bootstrapped‐pseudobulk aging clocks (correlations 0.78–0.89, and MAEs 8.5–11.2 years) (Figure 3c; Data S6m–r and S7, Supporting Information). However, the cell‐type‐specific simple pseudobulk aging clocks, with the exception of OPCs, performed relatively poorly with correlation coefficients as low as 0.4 and MAE as high as 18 years in oligodendrocytes (Figure 3d; Data S6g–l and S7, Supporting Information), suggesting that the bootstrapped‐pseudobulk and the single‐cell approaches captured more variation, allowing for greater accuracy in age predictions.

Next, we evaluated non‐cell‐type‐specific pseudobulk aging clocks at the neuron level (based on aggregated gene expression data across inhibitory neurons and excitatory neurons), glia level (based on aggregated gene expression data across oligodendrocytes, astrocytes and OPCs) and across cells of all major cell types (based on aggregated gene expression) (Figure S3a; Data S6s–aa, Supporting Information). The bootstrapped‐pseudobulk clocks in all three cases made predictions with high positive correlation, with a correlation coefficient of 0.9 and MAE of 8 years in the all‐cells pseudobulk clock, for example (Figure S3b, Data S6s–aa and S7, Supporting Information). Like the cell‐type‐specific case, we observed that the bootstrapped‐pseudobulk aging clocks showed better performance in age prediction compared to the simple pseudobulk clocks. For instance, the glia‐level simple pseudobulk clock had a correlation coefficient of 0.2, while in the bootstrapped versions, the correlation coefficient was around 0.8 (Figure S3b, Data S6s–aa and S7, Supporting Information). This further suggests that bootstrapping prior to training leads to more accurate predictions, possibly by sampling a greater range of variation in the data. Additionally, the similar performance in the bootstrapped neuron and glia level clocks suggests that the age‐related transcriptomic changes can also be modelled across cells in the neuronal lineage and in the glial lineage. These results highlight the importance of capturing dynamic differences between cell types, which non‐cell‐type‐specific clocks, despite their high performance, fail to address.

In summary, the overall performance of the cell‐type‐specific aging clocks indicates that human brain aging can be accurately defined in each of the major cell types at the single‐cell level, based on single‐nuclei transcriptomic data from the human post mortem prefrontal cortex tissue. In addition, these results strongly suggest the differences in age‐related dynamics between cell types in the brain and the importance of studying aging at a cell‐type‐specific level.

### 2.4 Clock Genes Capture Cell‐Type‐Specific and Biologically Relevant Aging Pathways

While the clocks effectively predict age in the training datasets, it is important for the feature gene set to have biological relevance to aging. To check this, we examined the feature genes corresponding to each clock model, defined as those with non‐zero coefficients across all the training rounds (Data S5, Supporting Information). In both the single‐cell and bootstrapped‐pseudobulk cell‐type‐specific clocks, we observed that most of the feature genes were unique to each cell‐type‐specific clock, while only a small proportion overlapped between them (Figure S4a–c and Data S5, Supporting Information)—further suggesting that the transcriptomic signatures captured by the clock models are largely cell‐typespecific. Notably, the total number of genes associated with the cell‐type‐specific clock models was consistently lower in the bootstrapped‐pseudobulk approach when compared to the single‐cell approach (Figure S4b–f, Supporting Information), possibly because single‐cell data captures greater variation at the individual cell level.

In all the different clock models, we observed feature genes with both positive and negative average coefficients (Figure S4d–f and Data S5, Supporting Information), suggesting that the clocks capture genes with both an increasing and decreasing age‐related trend. With the gene over‐representation test of the various single‐cell‐clock feature genes, we found several age‐related pathways to be enriched (Figure S4g and Data S9, Supporting Information). In microglia, terms related to cytokine production and inflammatory response were enriched for feature genes with a positive coefficient (Data S9b, Supporting Information), suggesting an association with increased inflammatory response, as was observed in the differential gene expression analysis (Figure 2c). Genes such as *SLC1A3*, *FOXP1*, and *MS4A6A*, associated with inflammatory response, and microglia in aging and Alzheimer's disease, had positive average coefficients among the microglia clock feature genes (Data S5c,o, Supporting Information). Additionally, the homeostatic microglia marker, *CX3CR1*, showed a negative average coefficient, consistent with our findings in the differential gene expression analysis. In the clocks trained on oligodendrocytes, astrocytes, OPCs, and inhibitory neurons, feature genes with negative average coefficients were enriched for terms related to synaptic transmission (Figure S4g, Data S9e,f,h,j, Supporting Information). Interestingly, in the excitatory neuron clock, synaptic transmission terms were enriched among genes with positive average coefficients (Figure S4g and Data S9i, Supporting Information). This aligns with prior reports showing that synaptic function is particularly vulnerable in the aging prefrontal cortex.^[^ ^18^, ^32^, ^33^, ^34^, ^35^ ^]^ The involvement of glial cell clocks‐ particularly oligodendrocytes and astrocytes in synaptic pathways‐ may reflect age‐related impairment of their supportive roles in neuronal communication. Furthermore, gene over‐representation analysis of feature genes from the glial and neuronal non‐cell‐type specific clocks showed similar pathway enrichment (Data S9l,m, Supporting Information), suggesting that while aging trajectories differ between cell types, certain biological functions—such as synaptic signaling—are commonly affected across the aging brain.

Overall, the only common feature gene we observed among all the cell‐type‐specific clocks was *FKBP5*, which had a positive average coefficient (Data S5, Supporting Information), suggesting an increasing trend. Spearman's correlation test *of FKBP5* expression across all the cell types further confirmed the predicted trend with a significant positive correlation (Correlation coefficient = 0.7) (Figure S4h, Supporting Information). *FKBP5* has been associated with aging by showing altered cognitive function in older adults and modulating brain connectivity.^[^ ^36^, ^37^ ^]^

In summary, these results show that clock feature genes capture distinct, biologically relevant, cell‐type‐specific aging pathways, including several known aging biomarkers—highlighting the interpretability and physiological relevance of the models.

### 2.5 Validation of Cell‐Type‐Specific Aging Clocks on Independent Single‐Nuclei RNA Sequencing Datasets

To test the applicability of the aging clock models on independent datasets, we selected publicly available snRNA‐seq datasets containing neurotypical adult human post mortem prefrontal cortex cohorts from two recent studies that have a broad and continuous age range and come from different subregions of the prefrontal cortex^[^ ^18^, ^38^ ^]^ (Table S1, Supporting Information). In the Fröhlich et al. dataset,^[^ ^18^ ^]^ we used 33 control samples from the orbitofrontal cortex, which ranged in age from 26 to 84 years with a median PMI of 29.75 (6.5–50) h (Table S1, Supporting Information). From the Velmeshev et al. dataset,^[^ ^38^ ^]^ we used 12 adult frontal cortex samples, aged 19 to 54 years, with a median PMI of 16.5 (6–27) h (Table S1, Supporting Information). In all cases, we focused on the major cell types as used above. In addition, to verify whether the datasets were comparable, we performed label transfer and projected the Uniform Manifold Approximation and Projection (UMAP) structure using common anchors between the training and the independent datasets (Figure S5 and Data S10, Supporting Information). We found that the clusters corresponding to various cell types in the UMAPs of both Fröhlich et al.^[^ ^18^ ^]^ (Figure S5a, Supporting Information) and Velmeshev et al.^[^ ^38^ ^]^ (Figure S5b, Supporting Information) aligned with those of the training dataset. Further, the predicted labels had high prediction scores for the matching original labels of all the cell types (Figure S5c,d, Data S10a,c, Supporting Information), with 90.5% matches in the Fröhlich et al. dataset^[^ ^18^ ^]^ (Data S10b, Supporting Information), and 98% matches in the Velmeshev et al. dataset^[^ ^38^ ^]^ (Data S10d, Supporting Information), suggesting that majority of the cells were comparable. We used the label predictions only for the above comparison and used the original annotations when applying the clocks on the external datasets.

**Figure 4:**
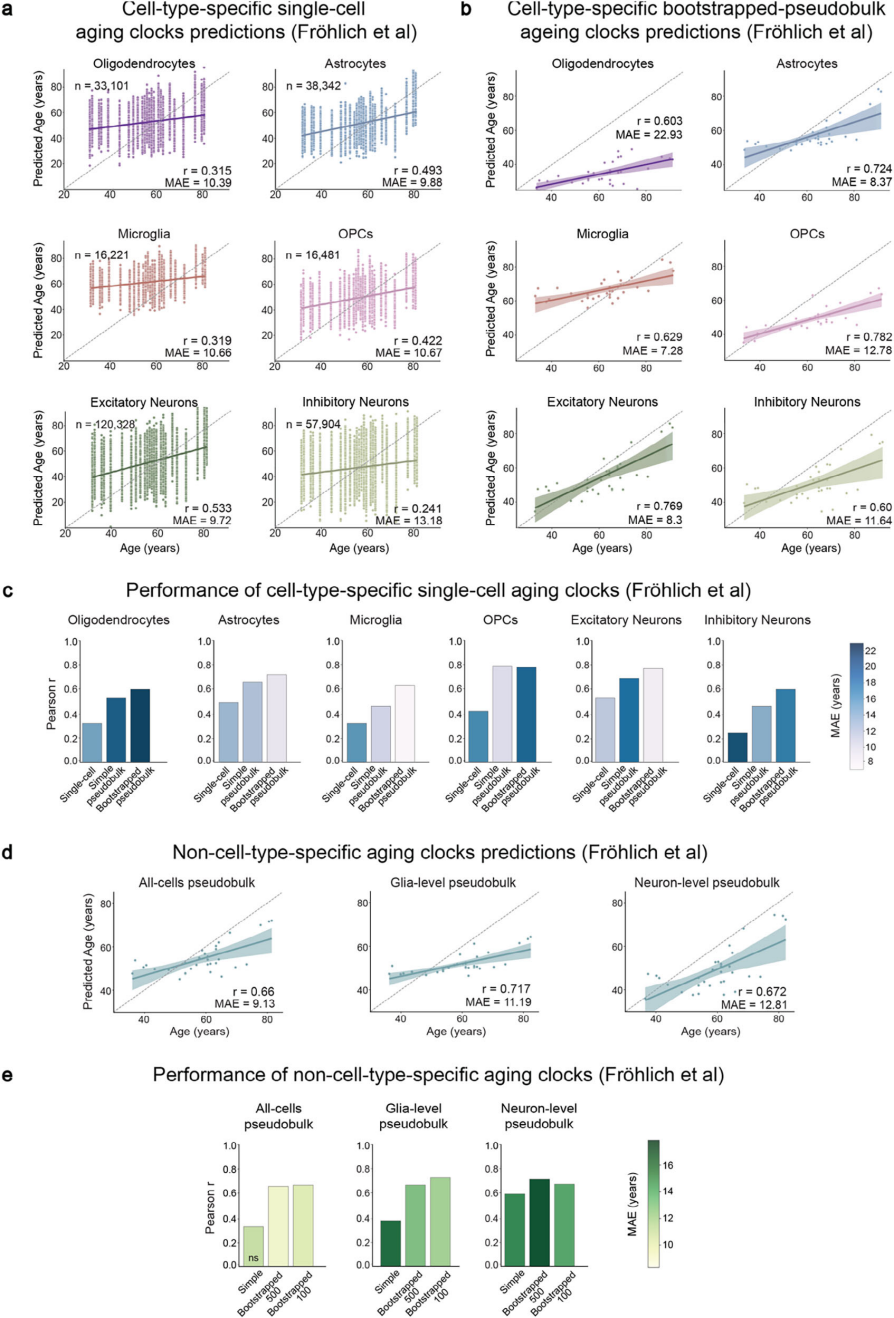
Validation of aging clocks on Fröhlich et al. dataset.^[^ ^18^ ^]^ All plots show results from the validation of various aging clocks in the Fröhlich et al. dataset.^[^ ^18^ ^]^ a, b) Relationship between chronological age and predicted age in each cell, upon using (a) the cell‐type‐specific single‐cell aging clock, and (b) the cell‐type‐specific bootstrapped‐pseudobulk aging clock approaches. c) Bar plots showing Pearson's correlation coefficients and mean absolute errors (MAE, represented by the intensity of blue colour in the bars) of each of the cell‐type‐specific approaches in each cell type. d) Relationship between the chronological age and the predicted age based on using the different non‐cell‐type‐specific pseudobulk clock approaches. e) Bar plots showing Pearson's correlation coefficients and mean absolute errors (MAE, represented by the intensity of green colour in the bars) of each of the non‐cell‐type‐specific approaches. All correlation tests were performed using stats.pearsonr function of SciPy with significance based on a *p*‐value < 0.05.

We applied the trained clocks to the different datasets and checked the correlation of the predictions with the chronological age of the donors using Pearson's correlation coefficient and the mean absolute error (MAE) of the predictions. In the Fröhlich et al. dataset,^[^ ^18^ ^]^ all clocks showed a statistically significant positive correlation between the predicted age and the chronological age. The level of correlation varied between the different cell types, with excitatory neurons showing the highest correlation and inhibitory neurons showing the lowest (**Figure** **4**a–c; Data S11 and S13, Supporting Information). The cell‐type‐specific single‐cell aging clocks predicted with low correlation coefficients between 0.22 and 0.54 and MAE between 7.6 and 10 years (Figure 4a,c; Data S11a–f and S13, Supporting Information), while the cell‐type‐specific bootstrapped‐pseudobulk aging clocks predicted with higher correlation coefficients between 0.56 and 0.78 and MAE between 7.1 and 15.6 years (Figure 4b,c; Data S11m–r and S13, Supporting Information). In the cohort of Velmeshev et al,^[^ ^38^ ^]^ the cell‐type‐specific single‐cell aging clocks showed statistically significant, but generally low correlations between 0.15 and 0.3 (Figure S6a,c, Data S12a–f and S13, Supporting Information), whereas the predictions of the cell‐type‐specific bootstrapped‐pseudobulk aging clocks showed no statistically significant correlation in any of the cell types except inhibitory neurons and OPCs (Figure S6b,c, Data S12m–r and S13, Supporting Information).

The non‐cell‐type‐specific bootstrapped‐pseudobulk aging clocks (for all cells, neurons, and glia levels) showed overall high positive correlations (r = 0.64–0.71) in the Fröhlich cohort (Figure 4d,e; Data S11s–y and S13, Supporting Information). In the Velmeshev cohort, on the other hand, interestingly, none except the neuron‐level clock showed a statistically significant correlation (Figure S6d,e, Data S12s–y and S13, Supporting Information).

The aging clocks depend on their feature gene sets for effective predictions. Given the dataset‐specific differences, such as in the number of genes detected per nucleus (Table S1, Supporting Information), we checked the expression of the clock feature genes in the external datasets to assess their potential influence on the varied performance. Across all the clock models and approaches, 95%–100% of the feature genes were found to be expressed in the dataset from Fröhlich et al.^[^ ^18^ ^]^ (Figure S7a, Supporting Information). In contrast, a lower proportion, ≈52%–85% of the genes, were expressed in the dataset from Velmeshev et al.^[^ ^38^ ^]^ (Figure S7a, Supporting Information), which may have possibly contributed to the lower performance.

Further, given that the clock models predict age based on the average regression coefficients of the feature genes across training rounds—with the sign of each coefficient indicating the direction of the gene's expression change with age—we examined how consistent these trends were across the datasets. Specifically, we calculated the proportion of genes that correlated with age in the same direction as those in the clock feature set (Data S14, Supporting Information). In the training dataset, 70%–99% of the genes significantly correlated in the same direction as the feature gene sets across all the clock models (Figure S7b, Supporting Information). On the other hand, in the Fröhlich et al.^[^ ^18^ ^]^ dataset, 40%–80% of the genes correlated in the same direction as the single‐cell clock feature sets, while it was 8 to 40% in the bootstrapped‐pseudobulk clocks (Figure S7b, Supporting Information). In the Velmeshev et al.^[^ ^38^ ^]^ dataset, the proportions were much lower, with only 15%–40% matching the trend in the single‐cell clocks, while almost none were significantly correlated in the bootstrapped‐pseudobulk clocks (Figure S7b, Supporting Information). The lower proportion of the feature gene set expression with age conforming to the clocks’ average coefficients may offer additional explanation for the lower performance in the Velmeshev et al.^[^ ^38^ ^]^ dataset.

In summary, we found that the cell‐type‐specific bootstrapped‐pseudobulk clocks showed a higher correlation and lower MAE in comparison to the single‐cell clocks, possibly due to lower transcriptional noise. Importantly, our analysis revealed that performance differences across external datasets are partly explained by variation in the expression and age‐correlation patterns of the clock feature genes. Despite these differences, the bootstrapped‐pseudobulk clocks demonstrated the ability to predict biological age at a cell‐type‐specific level in independent datasets, supporting their robustness and generalizability.

### 2.6 Aging Clocks Suggest Age Acceleration in Neurological Disorders

Age‐related changes have previously been reported to accelerate in various neurological disorders.^[^ ^17^, ^18^, ^39^, ^40^, ^41^, ^42^, ^43^, ^44^, ^45^ ^]^ Given that our clocks capture age‐related dynamics at a cell‐type‐specific level, we investigated whether they could also detect age acceleration in data from individuals diagnosed with age‐associated neurological diseases. We applied our various clocks to two publicly available snRNA‐seq datasets derived from post mortem prefrontal cortex tissue of individuals diagnosed with schizophrenia^[^ ^18^ ^]^ and Alzheimer's disease,^[^ ^46^ ^]^ respectively (Data S15 and S17, Supporting Information). Further, we compared the age acceleration^[^ ^47^ ^]^ in the disease cohorts versus control samples within each dataset (Data S16 and S18, Supporting Information).

**Figure 5:**
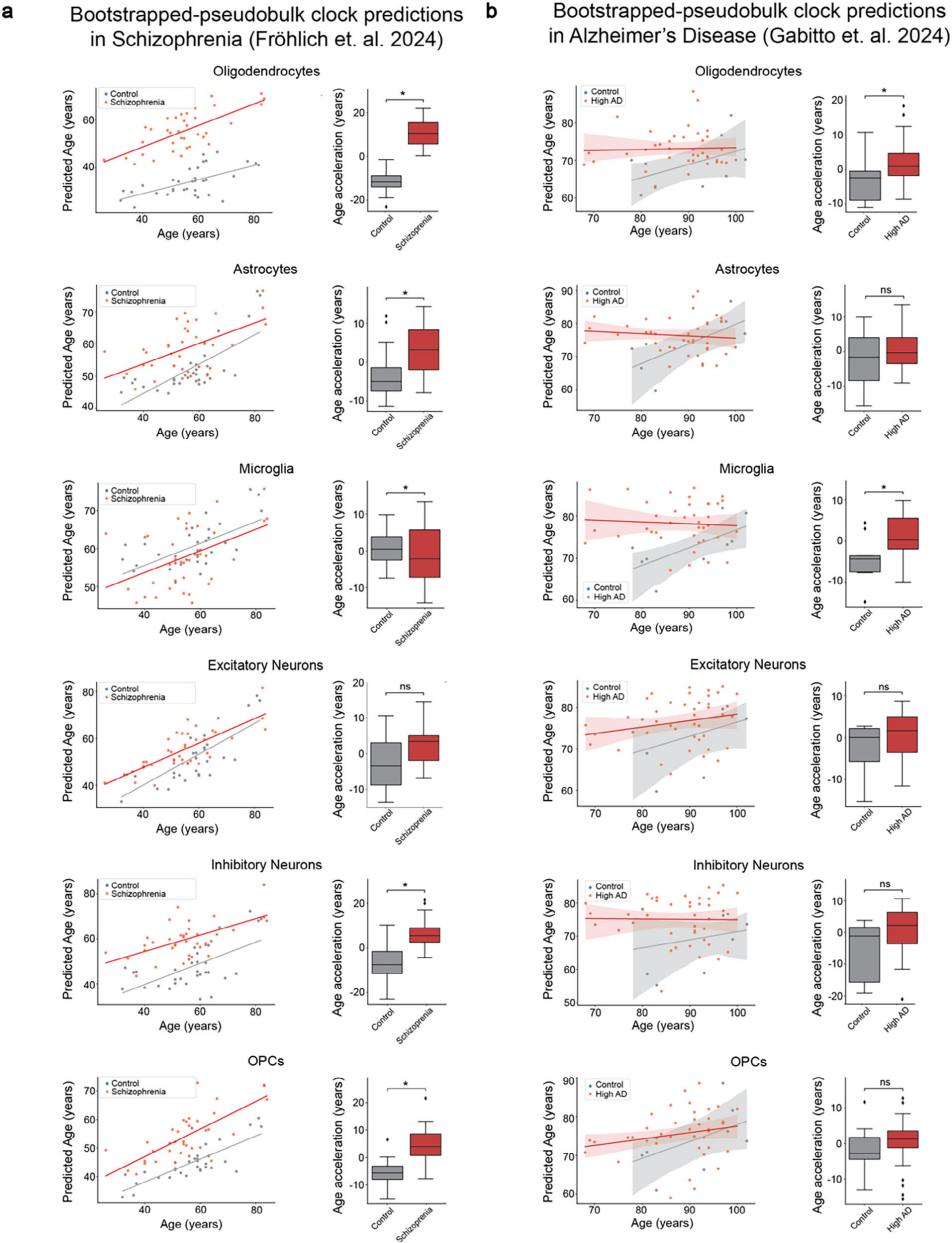
Age acceleration in Alzheimer's disease and schizophrenia. All plots show results from the application of bootstrapped‐pseudobulk cell‐type‐specific aging clocks in each of the major cell types in snRNA‐seq data of post mortem prefrontal cortex tissue from Fröhlich et al.^[^ ^18^ ^]^ and Gabitto et al.^[^ ^46^ ^]^ a) Left‐Relationship between chronological age and predicted age in schizophrenia (red) and control samples (grey). The line was computed with seaborn.regplot function represents the regression line fitted to the points of the given condition. Right‐Box plots showing the age acceleration in control samples (grey) and schizophrenia (red). b) Left‐Relationship between chronological age and predicted age in High‐AD (red) and control samples (grey). The line was computed with seaborn.regplot function represents the regression line fitted to the points of the given condition. Right‐Box plots showing the age acceleration in control samples (grey) and High‐AD samples (red). ^*^ *P* < 0.05; Generalized Linear Model was used to determine the significance of the difference between the age acceleration of control and disease samples (Experimental Section).

In the schizophrenia cohort (Fröhlich et al.^[^ ^18^ ^]^), all cell‐type‐specific clocks — except those for microglia, and the bootstrapped‐pseudobulk approach in excitatory neurons — showed statistically significant age acceleration in patients compared to controls (**Figure** **5**a; Figure S8a and Data S16, Supporting Information). Notably, the microglia clock predictions showed a deceleration of age relative to the controls.

In the Alzheimer's disease cohort (Gabitto et al^[^ ^46^ ^]^), the oligodendrocyte and microglia clocks consistently indicated significant age acceleration in High‐AD samples compared to the controls across all approaches (Figure 5b; Figure S8c and Data S18, Supporting Information). Other cell‐type‐specific clock predictions showed trends toward accelerated age, but the differences were not statistically significant (Figure 5b; Figure S8c and Data S17 and S18, Supporting Information).

All the non‐cell‐type‐specific clocks revealed significant age acceleration in the schizophrenia samples but not in the Alzheimer's disease samples (Figure S8b,d and Data S16 and S18, Supporting Information).

In summary, these findings demonstrate the applicability of our aging clocks to external datasets and highlight cell‐type‐specific patterns of age acceleration in neurological disorders, underscoring their relevance and potential as tools for studying disease‐associated aging.

## 3 Discussion

In this study, we have generated a high‐quality snRNA‐seq dataset of post mortem human brains from a variety of prefrontal cortex subregions, with short PMIs and a broad age range spanning all three age groups of adulthood. The dataset has been used to develop human single‐nucleus‐based transcriptomic aging clocks for each of the major cell types in the human prefrontal cortex, which can closely predict age at a cell‐type‐specific level. The clocks predict age in all the major cell types, with the best performance in the bootstrapped‐pseudobulk aging clocks, where for example in the OPCs, microglia and excitatory neuron clocks have correlation coefficients of around 0.9, and in the single‐cell clocks of the same cell types with almost equal accuracy with correlation coefficients around 0.8, in the test samples of the training dataset.

Epigenetic clocks have previously been applied to the brain^[^ ^15^, ^48^, ^49^, ^50^, ^51^, ^52^ ^]^ and make predictions with high accuracy. However, they do not capture the cell‐type‐specific information that is important for the brain, given its wide range of cell types^[^ ^53^ ^]^ and cell‐type‐specific vulnerabilities.^[^ ^54^ ^]^ While cell‐type‐specific epigenetic clocks have been developed by using bulk brain tissue, sorted brain cells, and deconvolution algorithms, such methods have limitations (e.g., lower cell‐type resolution) when compared to the analysis of directly measured single cells.^[^ ^55^ ^]^ Although single‐cell DNA methylation approaches exist and single‐cell DNA methylation clocks have been developed for other organ systems,^[^ ^10^, ^11^ ^]^ high cost, high sparsity, low‐throughput, and low and random coverage in combination with the binary nature of the data pose major limitations for the development of aging clocks and their applicability.^[^ ^56^, ^57^, ^58^ ^]^ In this context, snRNA‐seq offers higher coverage, is scalable and high‐throughput at a lower cost,^[^ ^59^ ^]^ and its quantitative nature makes it more feasible for the development of cell‐type‐specific aging clocks. Further, the use of cell‐type‐specific transcriptomic clocks is more feasible given the high prevalence of single‐nucleus transcriptomics in the study of human brain aging and neurodegenerative diseases.

It is important to note that while both single‐cell and bootstrapped‐pseudobulk clocks showed high correlations in the training dataset, their performance in independent datasets was lower and varied. For example, the Velmeshev et al.^[^ ^38^ ^]^ post mortem samples originated from across the frontal cortex, which broadly encompasses the prefrontal cortex. Such regional generality could have introduced an increased variability in the cell‐type‐specific transcriptomic aging profiles. Additionally, the dataset included several middle‐aged samples, where the direction of changes could be more complex when compared to the remaining age groups.^[^ ^60^ ^]^ Further, given the dependence of the clock models on the feature gene sets, the high number of missing clock features may have also hampered the performance of the clocks. Overall, the differences in performance may have been due to variations in sample preparation method, tissue quality, PMI, quality control thresholds, brain regional specificity, and sequencing. Establishing thresholds for the minimum number of genes or cells required for optimal clock accuracy may help to better understand such differences. In addition, the donors in the training dataset were of limited geographical origin, so the variations were under‐represented. Although increasing the sample size with equal representation of different ethnicities and sexes may help to mitigate the above issue, the availability of good‐quality post mortem tissue in adulthood in such large numbers is a major challenge. The single‐cell clocks showed particularly poor performance, whereas the bootstrapped‐pseudobulk clocks showed much better performance; for example, excitatory neuron and OPC clocks made predictions with correlation coefficients close to 0.8. This falls in line with previous observations in Buckley et al.^[^ ^4^ ^]^ and suggests that aggregating and bootstrapping the data may have overcome the transcriptomic noise associated with snRNA‐seq. There is an urgent need to set standards and define the minimum criteria for further cell‐type‐specific single‐cell transcriptomic clock development.

In summary, we describe here a human brain aging snRNA‐seq dataset that has allowed us to construct transcriptomic clocks for all major cell types of the human brain. In age‐related neurological disorders, such as Alzheimer's disease and schizophrenia, several age‐related changes are often accelerated.^[^ ^39^, ^40^, ^41^, ^42^, ^43^, ^44^, ^45^, ^48^, ^49^, ^50^, ^51^, ^61^ ^]^ Our clocks reveal widespread acceleration of aging across nearly all cell types in the schizophrenia patient samples. In Alzheimer's disease, the clocks show significant age acceleration, specifically in oligodendrocytes and microglia from High‐AD samples. The variable performance of the clocks in other cell types in Alzheimer's disease may reflect the influence of more specific cellular subtypes within these populations. This highlights the need to further refine aging clocks with improved resolution for specific cell subtypes, cell states, and brain regions. Such advancements could offer critical insights into how aging contributes to cellular vulnerability and disease progression in the human brain.

## 4 Experimental Section

### Ethics Statement

This study adhered to the principles outlined in the Declaration of Helsinki and the Ethical Rules for Using Human Tissues for Medical Research in Budapest, Hungary (HM 34/1999). Activity of Human Brain Tissue Bank, Semmelweis University has been authorised by the Committee of Science and Research of Ethics of the Ministry of Health, Hungary (189/KO/02.6008/2002/ETT) and the Regional Committee of Science and Research Ethics (No.32/1992/TUKEB). All experiments involving human post mortem samples described in this study were conducted under the ethical approval number (IV/2627‐ 1 /2021/EKU).

### Human Post Mortem Prefrontal Cortex Tissue

Human post mortem brain tissue samples (Table 1) were obtained from the Human Brain Tissue Bank (Semmelweis University, Budapest, Hungary). The tissue samples were collected from 31 individuals who were not diagnosed with any psychiatric disorder or neurodegenerative disorder. The brains were removed with a PMI of 2–12 h, dropped frozen using dry ice, and stored at −80 °C until further dissection. Samples were punched out from the lateral surface of the prefrontal cortices of the diseased subjects using specialized microdissection needles with 8‐ and 15‐mm internal diameters. While most of the samples were dissected from the ventrolateral prefrontal cortex, some originated from the dorsolateral prefrontal cortex and from the middle frontal gyrus (the dorsal part of which belongs to the dorsolateral prefrontal cortex and its ventral part to the ventrolateral prefrontal cortex). The dissected cortical tissue pellets included both gray and white matter portions within the gyrus. Samples were collected in 1.5 mL Eppendorf tubes and stored at −80 °C until further use. Throughout the microdissection procedure, the tissue samples were kept frozen.

### Single‐Nuclei Isolation

The nuclei isolation from the frozen post mortem brain tissue was performed as described previously.^[^ ^62^, ^63^ ^]^ Briefly, the tissue was thawed and dissociated in ice‐cold lysis buffer [0.32 mol L^−1^ sucrose, 5 mmol L^−1^ CaCl_2_, 3 mmol L^−1^ MgAc, 0.1 mmol L^−1^ EDTA, 10 mmol L^−1^ Tris‐HCl (pH 8.0), and 1 mmol L^−1^ dithiothreitol] using a 1 mL tissue douncer (Wheaton). The homogenate was carefully layered on top of a sucrose solution [1.8 mol L^−1^ sucrose, 3 mmol L^−1^ MgAc, 10 mmol L^−1^ Tris‐HCl (pH 8.0), and 1 mmol L^−1^ dithiothreitol] before centrifugation at 30 000 × g for 2 h and 15 min. After supernatant removal, the pelleted nuclei were softened for 10 min in 50 µL of nuclear storage buffer [15% sucrose, 10 mmol L^−1^ Tris‐HCl (pH 7.2), 70 mmol L^−1^ KCl, and 2 mmol L^−1^ MgCl_2_] before being resuspended in 300 µL of dilution buffer [10 mmol L^−1^ Tris‐HCl (pH 7.2), 70 mmol L^−1^ KCl, and 2 mmol L^−1^ MgCl_2_] and filtered through a cell strainer (70 µm). Nuclei were stained with Draq7 and run through fluorescence‐activated cell sorting (FACS Aria, BD Biosciences) at 4 °C at a low flow rate using a 100 µm nozzle (reanalysis showed >99% purity). 8500 nuclei were sorted and used for downstream applications (snRNA‐seq). Detailed protocol can be found at doi: https://doi.org/10.17504/protocols.io.5jyl8j678g2w/v1.

### Single‐Nucleus RNA Sequencing

Nuclei intended for snRNA‐seq (8,500 nuclei per sample) were directly loaded onto the Chromium Next GEM Chip G or Chromium Next GEM Chip K Single Cell Kit along with the reverse transcription master mix following the manufacturer's protocol for the Chromium Next GEM single cell 3′ kit (PN‐1000268, 10x Genomics), to generate single‐cell gel beads in emulsion. cDNA amplification was done as per the guidelines from 10x Genomics using 13 cycles of amplification. Sequencing libraries were generated with unique dual indices (TT set A) and pooled for sequencing on a Novaseq6000 using a 100‐cycle kit and 28‐10‐10‐90 reads.

### Single‐Nucleus RNA Sequencing Analysis

Raw base calls were demultiplexed to obtain sample‐specific FastQ files, and reads were aligned to the GRCh38 genome assembly using the *Cell Ranger* pipeline (10x Genomics Cell Ranger 7.0.0; RRID:SCR_017344)^[^ ^59^ ^]^ with default parameters and the include‐introns options set to true. The resulting matrix files were used for further analysis.

All downstream analysis was done using *R* (v4.3.1; RRID:SCR_001905) and the standard workflow of *Seurat* (v4.3.0; RRID:SCR_016341).^[^ ^64^ ^]^ Nuclei with read counts between 1,200 and 100,000, gene counts between 800 and 12,000, and less than 5% mitochondrial transcripts were included for the analysis. After log‐normalization and scaling, the data were integrated using *Harmony* (v0.1.1; RRID:SCR_022206^)^,^[^ ^65^ ^]^ where both sequencing batches and individual samples were regressed out, and the first 39 principal components were chosen. Clusters resolved at a resolution of 0.2 were annotated based on canonical cell type markers of the human prefrontal cortex. Further, any doublets or multiplets detected using *scDblFinder* (v1.13.10; RRID:SCR_022700)^[^ ^66^ ^]^ were removed. Differential gene expression analysis was done using the *FindMarkers* function and the Wilcoxon Rank Sum Test, between different age groups, namely, old versus young, old versus middle‐aged, and middle‐aged versus young, followed by Benjamini–Hochberg correction for multiplicity. Genes with adjusted *p* value < 0.05 and absolute log two‐fold‐change greater > 0.5 were considered significant. Mitochondrial genes and sex genes were excluded from further downstream analysis. Enrichment of GO terms corresponding to significantly differentially expressed genes was tested with the gene over‐representation test using the *ClusterProfiler* package (v4.8.2; RRID:SCR_016884), with Benjamini‐Hochberg correction for multiplicity.

### Aging Clock Models

Chronological age was predicted based on the log‐normalized gene expression values using the Python implementation of the Glmnet algorithm for the ElasticNet regression model.^[^ ^30^, ^31^ ^]^ Mitochondrial and sex‐related genes were removed from the data, resulting in 35,578 genes in total to use as features. Separate models were trained directly on the cell data of the six major cell types, yielding cell‐type‐specific single‐cell age prediction models (i.e., aging clock) for oligodendrocytes, astrocytes, microglia, OPCs, excitatory neurons, and inhibitory neurons. To prevent information leakage between training and test data, the age prediction models were trained and tested with donor‐level 5‐fold cross‐validation (i.e., in one iteration, a model was trained using cells of 80% of the donors and predicted the age of cells of the remaining 20% of the donors). Different alpha parameter values (alpha = 0, 0.5, and 1) were experimented with and selected the models trained with alpha = 0.5 due to their best overall performance (also considering external performance) of predicting the age of single cells. Performance was measured by Pearson's correlation coefficient (r) between the chronological and predicted age, as well as by mean absolute error (MAE) of the predictions (Data S3, Supporting Information).

Besides the cell‐type‐specific single‐cell aging clock approach, cell‐type‐specific pseudobulk aging clocks were also developed using generated pseudobulk samples in two ways: i) simple pseudobulk where gene expressions were averaged over all cells of a given cell type of a given donor, resulting in one pseudobulk sample for each donor and cell‐type; and ii) bootstrapped‐pseudobulk where 100 samples were generated for each donor and cell‐type by randomly sampling and averaging a given number of cells (for oligodendrocytes: 200, astrocytes: 50, microglia: 50, OPCs: 50, excitatory neurons: 100, and inhibitory neurons: 100). The number of cells sampled was determined so that at least 80% of the donors have more cells available in the data to ensure variability in the bootstrapped‐pseudobulk samples. Then, similarly to the single‐cell approach, separate models were trained for each cell type with donor‐level 5‐fold cross‐validation, and with different values for parameter alpha (alpha = 0, 0.5, and 1). The predicted age of a donor given by the cell‐type‐specific bootstrapped‐pseudobulk aging clocks was calculated by the average prediction of the donor's 100 pseudobulk samples for the given cell type.

Non‐cell‐type‐specific pseudobulk aging clocks were also developed at the glia‐level, neuron‐level, and for all cells (simple and bootstrapped). The glia‐level clocks were based on oligodendrocytes, astrocytes, and OPCs; the neuron‐level clocks were based on excitatory and inhibitory neurons; and the all‐cells clocks used all cells from the six major cell types. Both simple and bootstrapped‐pseudobulk approaches were tested for the three levels. For the simple pseudobulk approach, samples were generated by averaging the gene expressions over all cells of the given cell types (neuron, glia, and all cells) of a donor. For the bootstrapped approach, 100 samples per donor were generated by taking the average over *k* randomly sampled cells of the given level. The approach was tested with k = 500 and 100 cells. In total, nine types of non‐cell‐type‐specific aging clocks (glia‐level/neuron‐level/all‐cells by simple, bootstrapped with 100 cells, and bootstrapped with 500 cells approach) were tested. The training and validation of the clocks were performed in the same way as described previously.^[^ ^12^ ^]^

In addition to the Glmnet algorithm, the decision tree‐based ExtraTrees Regressor model was also tested, implemented via the scikit‐learn Python package. However, due to its lower predictive performance for age estimation, the Glmnet models were relied on for this study. The summary of performance metrics of the ExtraTrees clock model can be found in Data S8 (Supporting Information).

### Naïve Mean Prediction Model

As a baseline model, the age of the test samples were predicted as the mean age of the training samples. The same sample generation, model training, and testing approach as described in “Aging clock models” was used. Further, instead of training an ElasticNet model in each round of cross‐validation on the training samples, the mean age of the samples was calculated and assigned as the predicted age to the test samples.

For simpler visualization, the average prediction of the 5 folds is shown in Figure 3 and Figure S3 (Supporting Information) as a reference prediction.

### Label Transfer and UMAP Projection

Using *Seurat* (v4.4.0; RRID:SCR_016341)^[^ ^64^ ^]^ and *R* (v4.3.1; RRID:SCR_001905),^[^ ^67^ ^]^ Seurat's recommended workflow for label transfer and UMAP projection was used. The processed snRNA‐seq data of the training dataset and the external datasets were subset to include only the major cell types. Additionally, in the Velmeshev et al.^[^ ^38^ ^]^ dataset, samples corresponding to adulthood, over 18 years of age, and originating from the frontal cortex were alone included. The raw counts from the Fröhlich et al.^[^ ^18^ ^]^ dataset were log‐normalized to keep them comparable to the training dataset. Further, the top 2,000 highly variable genes in the Fröhlich et al.^[^ ^18^ ^]^ dataset were identified using the *FindVariableFeatures* function, followed by re‐computing PCs and UMAP embeddings, using the *RunPCA* and *RunUMAP* functions, respectively.

With default parameters, the *FindTransferAnchors* function with PCA as reference reduction was used to find common anchors, where the training dataset with the first 18 PCs was used as reference, while the Fröhlich et al.,^[^ ^18^ ^]^ or Velmeshev et al.^[^ ^38^ ^]^ datasets were used as query. Using the derived anchors, the *TransferData* function was used to predict cell type annotations, and the *MapQuery* function was used to project UMAP embeddings in the query datasets. The predicted labels were only used for inferring the comparability of the datasets and did not replace the original annotations provided by the respective authors when applying the aging clocks.

### Aging Clock Validation on Independent External Datasets

To examine the generalizability of the proposed aging clocks, we applied them to two independent external datasets. A subset of the snRNA‐seq data of Velmeshev et al.^[^ ^38^ ^]^ namely, the frontal cortex samples of adult donors (age > 18 years) were used for external validation of the clocks, as well as orbitofrontal cortex snRNA‐seq data of the control samples of Fröhlich et al.^[^ ^18^ ^]^ In both cases, the processed gene expression data were utilized, and further processing of the samples was performed as described above in the section “Aging clock models”. Missing values were imputed by the average expressions of the missing genes of the training dataset, where the mean was calculated on the samples the clocks were trained on (e.g., on the bootstrapped samples for the bootstrapped clocks). Due to the 5‐fold cross‐validation described above, five regression models were generated for each clock. All five models were applied to each external sample, and their mean prediction was used for evaluation. The evaluation of the clocks on the independent datasets was done as described in “Aging clock models”.

### Correlation Analysis of Aging Clock Feature Genes in External Datasets

Using *R* (v4.3.1; RRID:SCR_001905)^[^ ^67^ ^]^ and *Seurat* (v4.4.0; RRID:SCR_016341),^[^ ^64^ ^]^ the single‐cell expression matrices in the different datasets were extracted, and the correlation between chronological age and clock‐selected feature genes of Single‐cell clocks was tested using Spearman's correlation test. Benjamini‐Hochberg correction was performed on the *p*‐values, and significance was measured at an adjusted *p*‐value < 0.05. For the clock‐selected feature genes of cell‐type‐specific bootstrapped‐pseudobulk clocks, the average expression was used across cells in each cell type per sample. While in the non‐cell‐type‐specific clocks, the expression was averaged across the different cell groups in each sample, i.e., across all major cell types for the all‐cells clock genes, across glial cells for the glia‐level clock genes, and across neurons for the neuron‐level clock genes.

### Application and Evaluation of Aging Clocks on Neurological Disorder Datasets

The most prominent aging clocks, namely, the single‐cell, cell‐type‐specific bootstrapped‐pseudobulk, all‐cells, glia‐level, and neuron‐level bootstrapped‐pseudobulk clocks with 100 cells, were applied to two neurological disorder datasets: the subset of the data of Fröhlich et al.^[^ ^18^ ^]^ of patients with schizophrenia and the subset of the data of Gabitto et al.^[^ ^46^ ^]^ of High‐AD and control samples. The raw expression counts were log‐normalized, and the clocks were applied as described in “Aging clock validation on independent external datasets”. Age acceleration of samples was calculated by fitting a least‐squares linear regression model on the predicted age with chronological age as the independent variable. Then, age acceleration was given by the difference between the predicted age and the regression line.^[^ ^47^ ^]^

Age acceleration of disease and control groups based on the single‐cell samples was compared using a Mixed Linear Model (dependent variable: age acceleration, independent variables: age, sex, disease, PMI, brain.ph for the Gabitto et al.^[^ ^46^ ^]^ data and additionally hemisphere for the Fröhlich et al.^[^ ^18^ ^]^ data) using the donors as the grouping variable to account for the batch effect caused by having multiple samples from each donor. For the other types of clocks, the disease and control groups were compared using a Generalized Linear Model with the above‐described dependent and independent variables. Then, the significance of the difference between the two groups was determined by the *p*‐value of the coefficient of the disease variable (0: control, 1: diseased) in the above models.

## Conflict of Interest

The authors declare no conflict of interest.

## Author Contributions

C.K., J.J., and K.P. contributed equally to this work. M.P. and É.R. provided the microdissected human brain samples used in the study. A.A., J.G.J., and D.A.M.A. carried out the snRNA‐seq experiments under the supervision of J.J. A.A.A. designed and visualized the figures. C.M. designed the single‐cell computational framework and analysed the data under the supervision of K.P., Y.S., and J.J. Y.S. and R.G. verified the analytical methods. E.Z.‐P. developed and applied the single‐cell transcriptomic clocks under the supervision of C.K. C.M. wrote the manuscript with support from K.P., C.K., and J.J., and with input from all authors. K.P. and C.K. conceived the study and were in charge of overall direction and planning. K.P., J.J., and C.K. supervised the project. All authors provided critical feedback and helped shape the research, analysis, and manuscript.

## Supporting information



Supporting Information

## Data Availability

All data needed to evaluate the conclusions in the paper are present in the paper and/or the Supplementary Materials section. The training dataset has been deposited in the Gene Expression Omnibus (GEO) superseries GSE291605. In case of the external validation and application datasets, the processed snRNA‐seq data of Velmeshev et al.^[^ ^38^ ^]^ can be accessed at https://cellxgene.cziscience.com/collections/bacccb91‐066d‐4453‐b70e‐59de0b4598cd, that of Fröhlich et al.^[^ ^18^ ^]^ is available under GEO superseries GSE254569, while the dataset of Gabitto et al.^[^ ^46^ ^]^ is available at https://sea‐ad‐single‐cell‐profiling.s3.amazonaws.com/index.html#PFC/RNAseq/. The supplementary data related to the manuscript along with the processed snRNA‐seq data, provided as RDS file containing Seurat object, can be accessed at https://doi.org/10.5281/zenodo.15188642. Clock models have been added to Data S5. The code for the single‐nuclei RNA sequencing analysis is available at https://github.com/chanmur/ppfctx_aging_snRNAseq_analysis_2025, and that for the aging clock part is at https://github.com/SZTAKI‐SU‐Rejuvenation‐Group/ppfctx_aging_snRNAseq_clock_2025.
