# Supplementary material for: Human Brain Cell‐Type‐Specific Aging Clocks Based on Single‐Nuclei Transcriptomics
Source: Adv Sci (Weinh). 2025 Aug 29;12(43):e06109. doi: 10.1002/advs.202506109 (PMC12631854; doi:10.1002/advs.202506109)
Supplement: Supplementary file 1 — Supporting Information [file ADVS-12-e06109-s001.pdf]

Supporting Information

Human brain cell-type-specific aging clocks based on single-nuclei transcriptomics

*Chandramouli Muralidharan[1, 2, 3], Enikő Zakar-Polyák[4, 5, 6], Anita Adami[1], Anna A. Abbas[2, 3], Yogita Sharma[1], Raquel Garza[1], Jenny G. Johansson[1], Diahann A. M. Atacho[1], Éva Renner[7], Miklós Palkovits[7], Csaba Kerepesi[4, 6 # *], Johan Jakobsson[1#], Karolina Pircs[2, 3, 1, 6 # *]*

All supplementary data related to the manuscript can be accessed at https://doi.org/10.5281/zenodo.15188642.

Supplementary Data

Following data are provided in a compressed .zip file

Supplementary Data 1: Sample information and distribution of cells from different cell types.

Supplementary Data 2: Gene expression in each cell type in the training dataset.

Supplementary Data 3: Differential gene expression analysis results from all major cell Types across all age-group comparisons.

Supplementary Data 4: Gene over-representation test results from all comparisons.

Supplementary Data 5: Aging clocks: regression models of each of the 27 aging clocks.

Supplementary Data 6: Predicted age from the testing rounds of each of the 27 aging clocks.

Supplementary Data 7: Summary of aging clock performances in the training dataset.

Supplementary Data 8: Summary of ExtraTrees Regressor model performances in the training dataset.

Supplementary Data 9: Gene over-representation test results for clock-selected feature genes

Supplementary Data 10: Label transfer prediction results in the external datasets.

Supplementary Data 11: Predicted age given by the 27 aging clocks in the control cohort of Fröhlich et al. dataset.

Supplementary Data 12: Predicted age given by the 27 aging clocks in Velmeshev et al. dataset.

Supplementary Data 13: Summary of aging clock performances in the external validation datasets.

Supplementary Data 14: Age correlation results of clock-selected feature genes in the training and the external validation datasets.

Supplementary Data 15: Predicted age given by the top aging clocks in the Schizophrenia cohort of Frohlich et al. dataset.

Supplementary Data 16: Summary of age acceleration comparison in the Fröhlich et al. dataset.

Supplementary Data 17: Predicted age given by the top aging clocks in the Gabitto et al. dataset.

Supplementary Data 18: Summary of age acceleration comparison in the Gabitto et al. dataset.

Table S1. Summary of sample details in the training and external datasets for major cell types after QC.

Attributes	Training Dataset	Fröhlich et al. Dataset	Velmeshev et al. Dataset
Number of Samples	31	33	12
Median Age (years)	54 (18 – 94)	57.5 (26 – 84)	29.5 (19 – 54)
Median PMI (hours)	4.5 (2 – 12)	29.75 (6.5 – 50)	16.5 (6 – 27)
Brain Region*	VLPFC, MFG, DLPFC	OFC	Frontal Cortex
Total number of nuclei	71,663	282,377	42,453
Median number of genes per nucleus	2,506<br>(801 – 11,625)	2,295<br>(361 – 5,750)	1,533<br>(319 – 9,113)
Median UMI count per nucleus	5,566<br>(1,201 – 99,998)	4,080<br>(500 – 14,204)	2,967<br>(359 – 93,098)

VLPFC – Ventrolateral Prefrontal Cortex, MFG – Medial Frontal Gyrus, DLPFC – Dorsolateral Prefrontal Cortex, OFC – Orbitofrontal Cortex

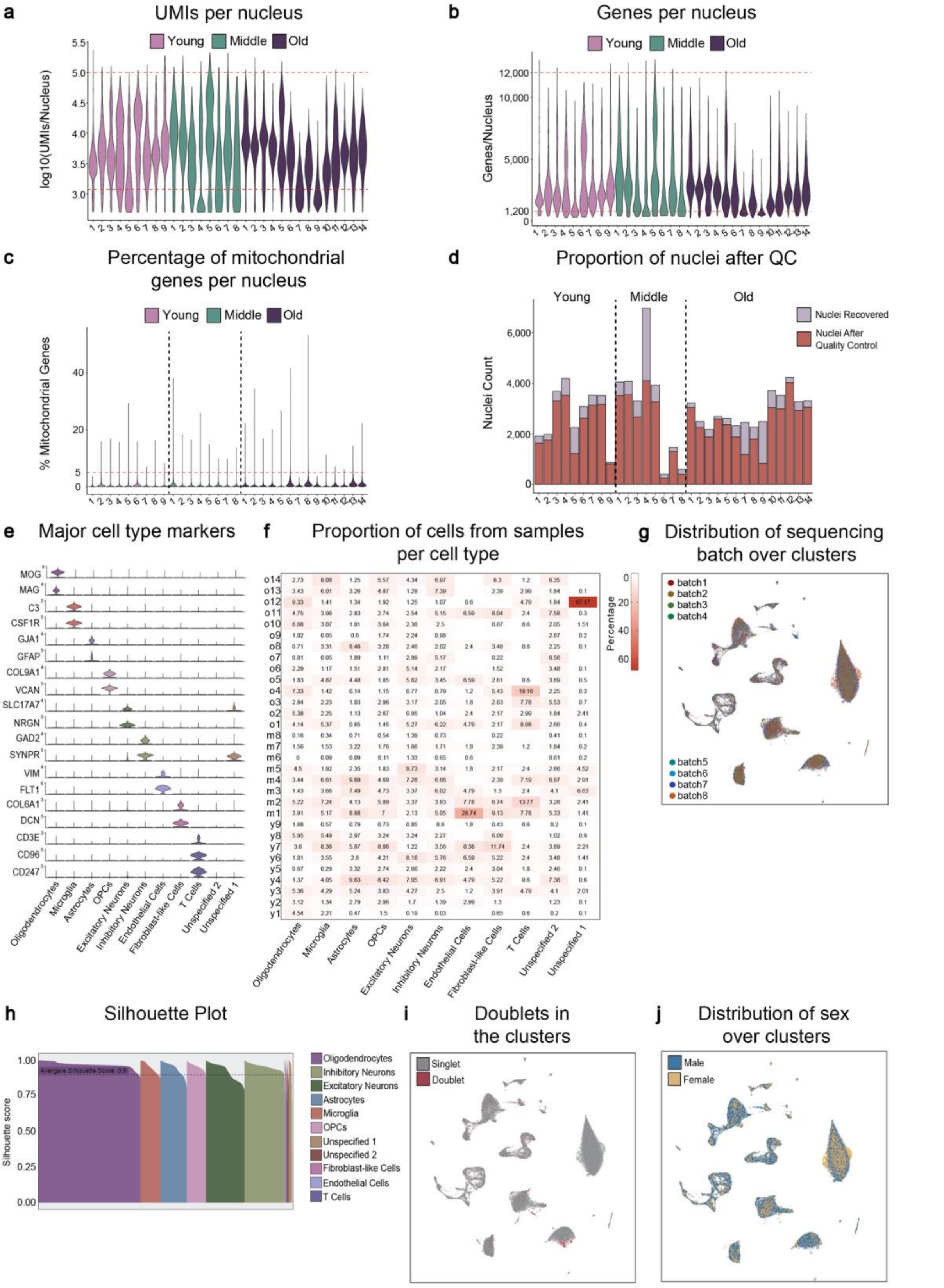

Figure S1. Quality and cell composition of the dataset. Related to Figure 1. (a) Violin plot showing the distribution of number of UMIs per nucleus per sample in log10 scale. (b) Violin plot showing the distribution of number of genes per nucleus per sample. (c) Violin plot

showing the distribution of percentage of mitochondrial genes per nucleus. (d) Bar plot showing the proportion of nuclei per sample after quality control. In a-d, the upper and lower limit of the range included are indicated by the red dashed lines. (e) Violin plots showing the expression of additional canonical markers corresponding to cell types in the prefrontal cortex. (f) Table showing the proportion of cells per sample in each cell type. (g) UMAP plot with annotation of sequencing batch in every cluster. (h) Plot showing the silhouette scores after Harmony integration. (i) UMAP plot with annotation of sex in every cluster. (j) UMAP plot showing annotation of predicted singlets and doublets.

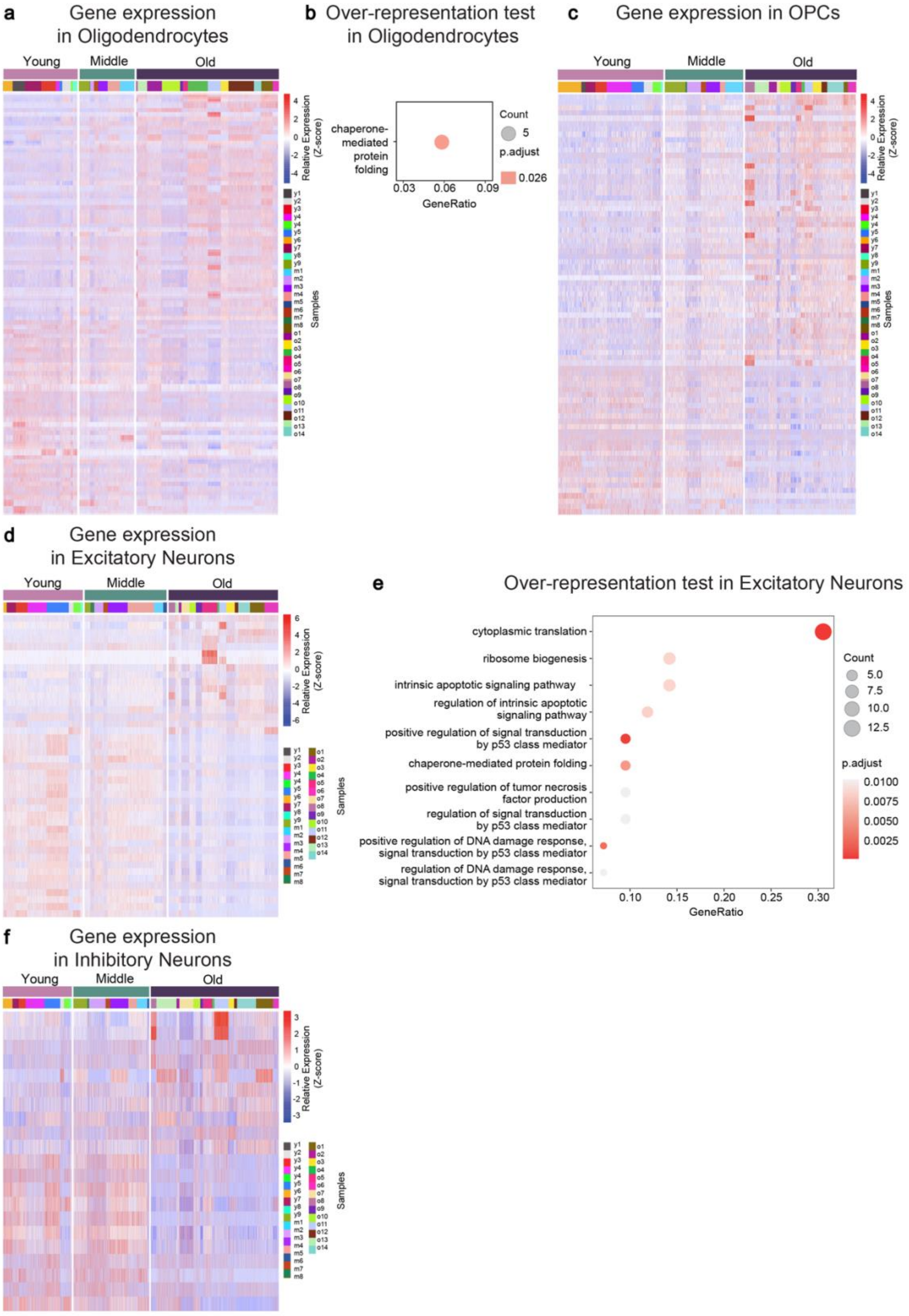

Figure S2. Age-related changes in various major cell types. Related to Figure 2. (a) Heatmap showing the expression of significantly differentially expressed genes in

oligodendrocytes when comparing old vs young age groups. (b) Plot showing gene ontology terms enriched from genes differentially expressed in old oligodendrocytes. (c) Heatmap showing the expression of significantly differentially expressed genes in OPCs when comparing old vs young age groups. (d) Heatmap showing the expression of significantly differentially expressed genes in excitatory neurons when comparing old vs young age groups. (e) Plot showing gene ontology terms enriched from genes differentially expressed in old excitatory neurons. (f) Heatmap showing the expression of significantly differentially expressed genes in inhibitory neurons when comparing old vs young age groups. *$p < 0.05$; ClusterProfiler's in-built test for gene-overrepresentation analysis along with Benjamini-Hochberg correction for multiplicity was used in b and e. Different colours below the young, middle and old ages bar represents cells originating from different individuals in a, c, d and f.

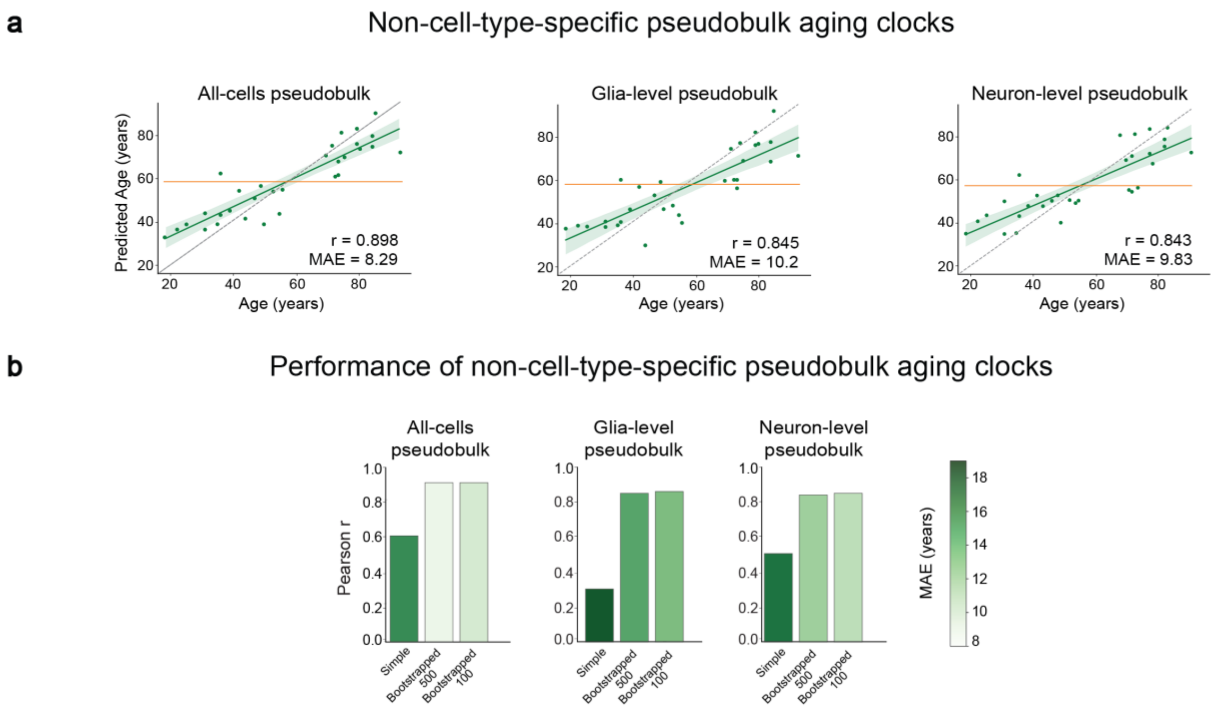

Figure S3. Development and evaluation of non-cell-type-specific bulk aging clocks. Related to Figure 3. (a) Relationship between the chronological age and the predicted age by using bootstrapped-pseudobulk aging clock approaches trained on all-cells, at the glia-level, and neuron-level, respectively. The horizontal line (orange) represents predictions from a naïve mean prediction model. (b) Pearson r and mean absolute error (MAE) from each pseudobulk clock approach. For each level (all-cells/glia-level/neuron-level), three versions are displayed: simple, bootstrapped with 500 cells, and bootstrapped with 100 cells, respectively. All correlation tests were performed using the stats.pearsonr function of SciPy with significance based on a False Discovery Rate (FDR) < 0.05.

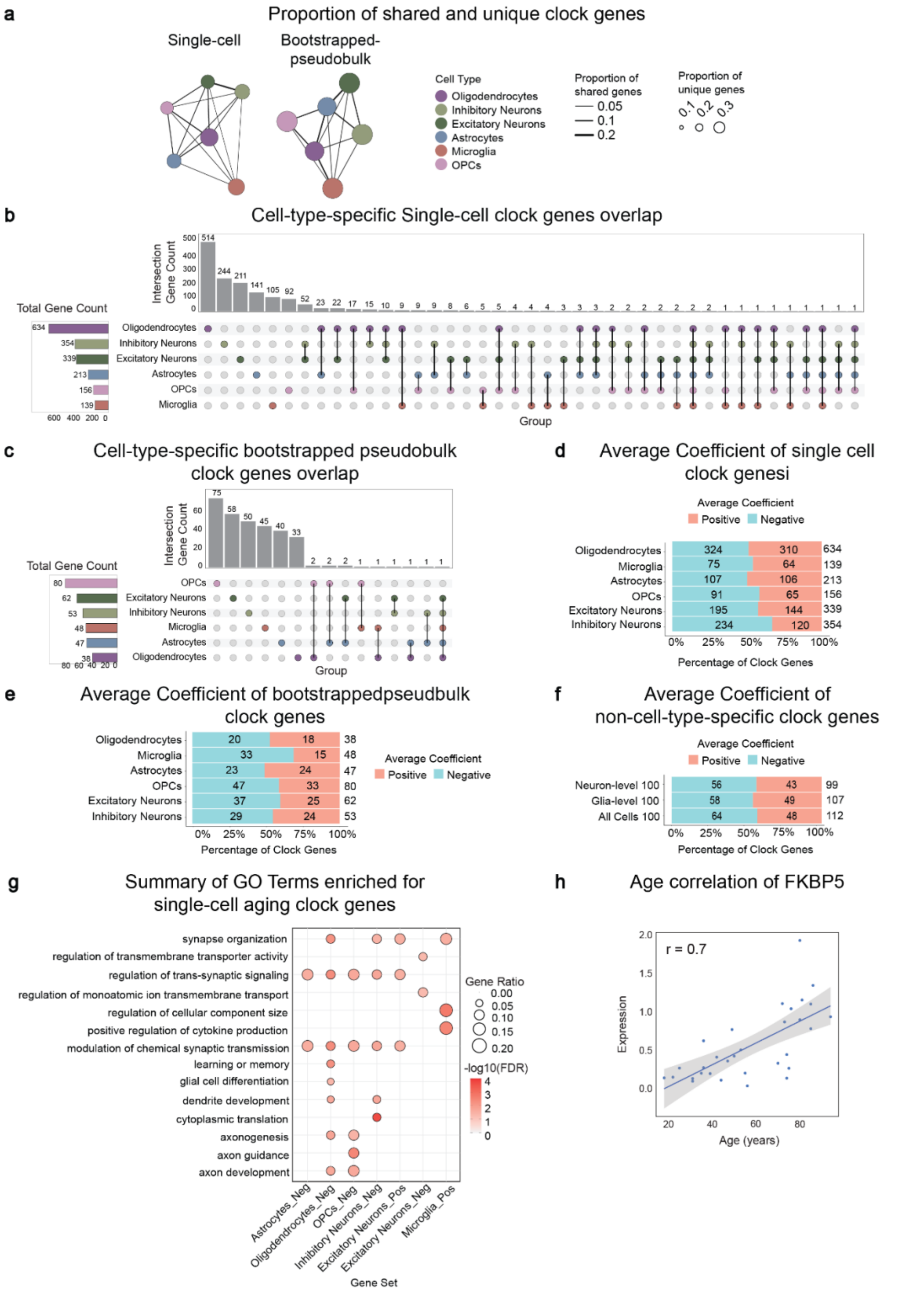

Figure S4. Clock-selected feature genes are associated with cell-type-specific aging-related pathways. Related to Figure 3. (a) Network plot showing the proportion of clock

feature genes unique and common to the different cell-type-specific clocks in the single-cell and the bootstrapped-pseudobulk approaches. (b-c) Upset plots showing the number and overlap of mutually exclusive clock-selected feature genes of the different cell-type specific ageing clock models in (b) the single-cell clock approach and (c) the bootstrapped-pseudobulk approach. (d-f) Bar plots showing the proportion and number of clock-selected feature genes with positive or negative average regression coefficient across all the training rounds in cell-type-specific (d) single-cell clocks, (e) bootstrapped-pseudobulk clocks and (f) the non-cell-type specific bootstrapped-pseudobulk clocks. (g) Plot summarising the gene ontology terms enriched for clock feature genes from each of the cell-type-specific single-cell aging clocks. Enrichment analysis was performed using ClusterProfiler's in-built over-representation test. (h) Correlation between the expression of FKBP5, pseudo-bulked per sample across the major cell types, and age of the samples. Correlation was estimated using a Spearman's correlation test. In (g-h) Benjamini-Hochberg correction for multiplicity was performed and significance was measured at an adjusted p-value < 0.05.

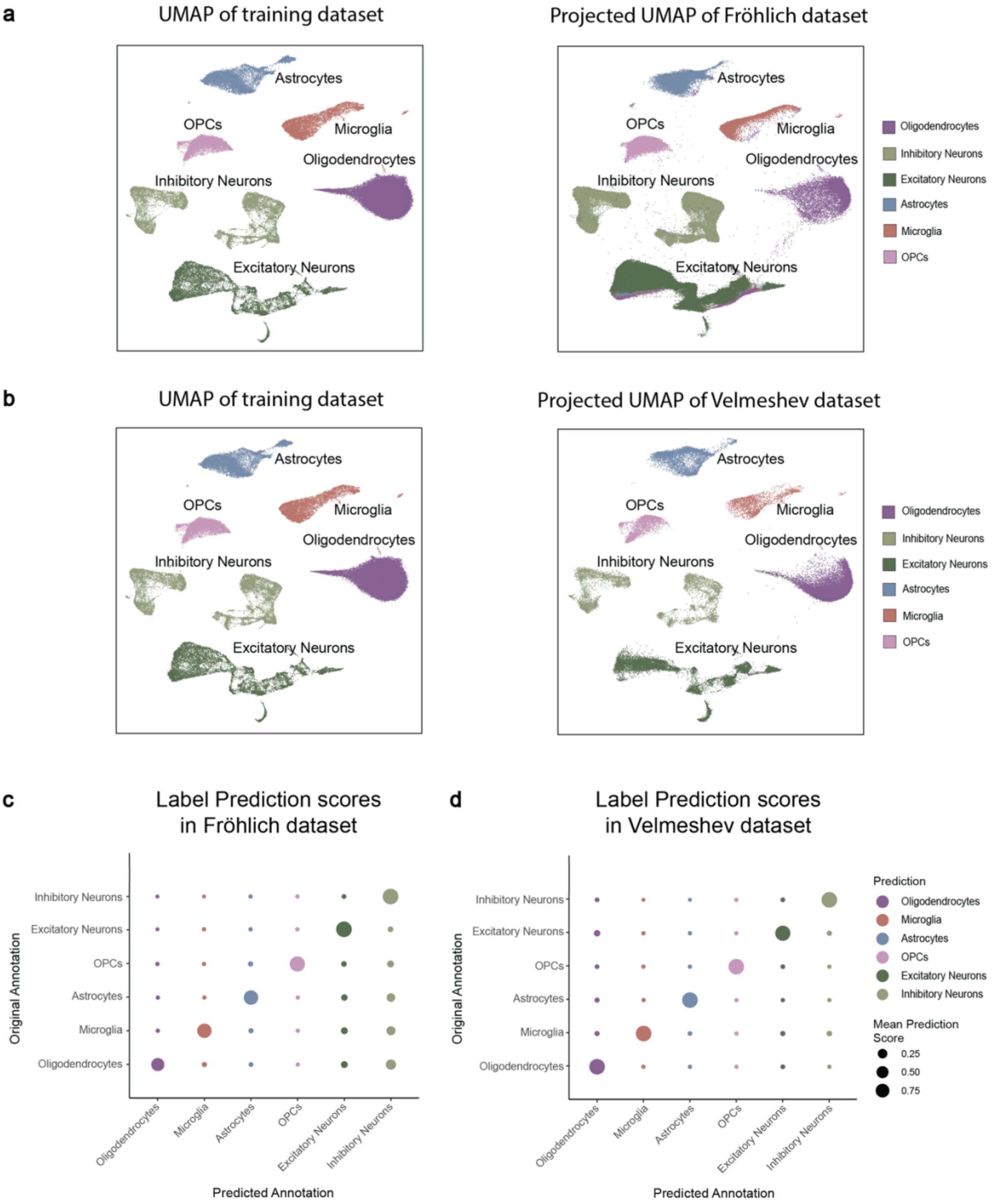

Figure S5. Comparison of cell type annotation and clustering between the training dataset and the validation datasets. Related to Figure 4. (a) UMAPs of training dataset as reference and its unimodal projection on Fröhlich et al[18] dataset. (b) UMAPs of training dataset as reference and its unimodal projection on the Velmeshev et al[32] dataset. (c) Bubble plot showing

the mean prediction score for different combinations of original annotations and predictions in the Fröhlich et al[18] dataset with the training dataset as reference. (d) Bubble plot showing the mean prediction score for different combinations of original annotations and predictions in Velmeshev et al[32] dataset with training dataset as reference.

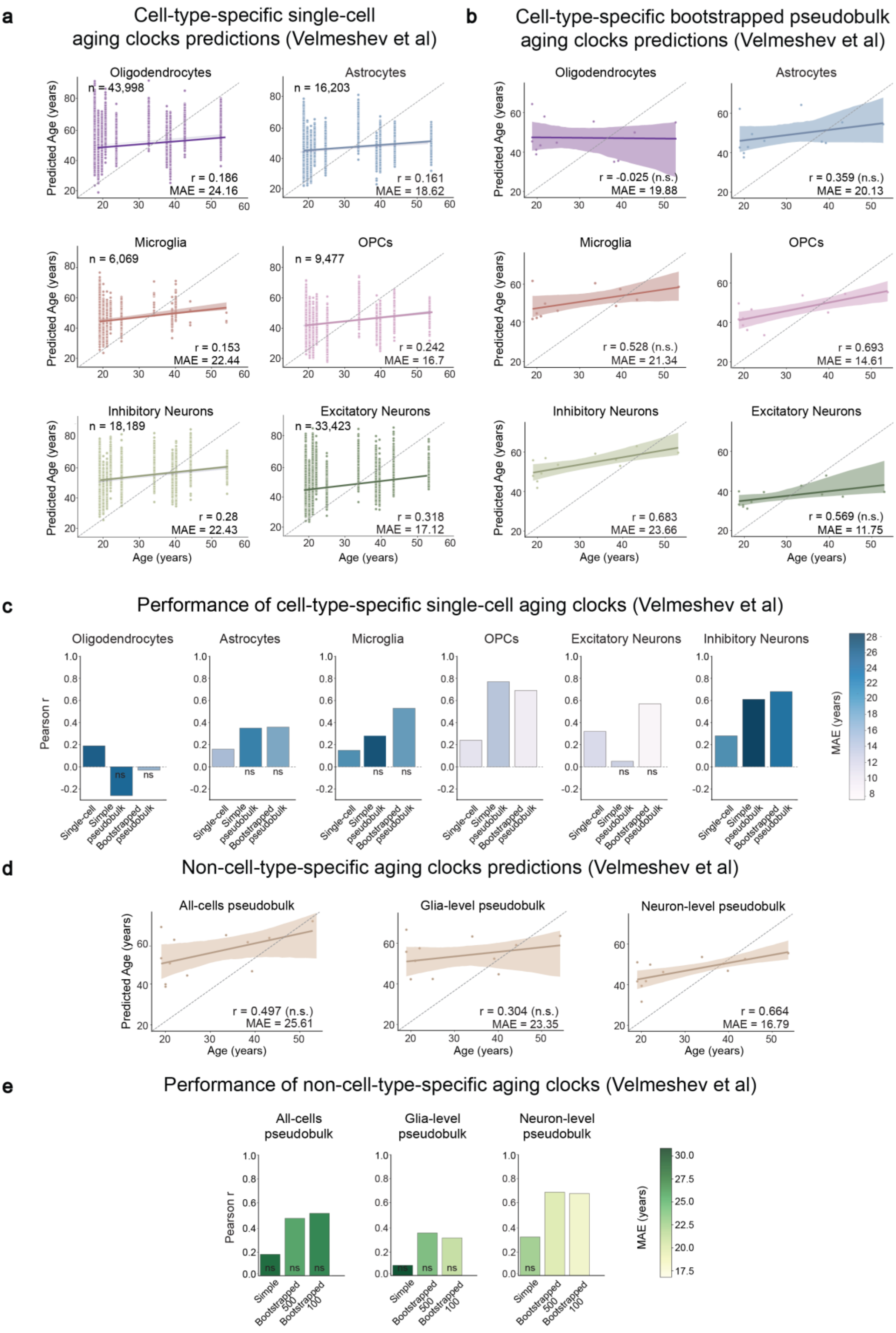

a
Cell-type-specific single-cell aging clocks predictions (Velmeshev et al)
Oligodendrocytes
n = 43,998
r = 0.186
MAE = 24.16
Astrocytes
n = 16,203
r = 0.161
MAE = 18.62
Microglia
n = 6,069
r = 0.153
MAE = 22.44
OPCs
n = 9,477
r = 0.242
MAE = 16.7
Inhibitory Neurons
n = 18,189
r = 0.28
MAE = 22.43
Excitatory Neurons
n = 33,423
r = 0.318
MAE = 17.12
Predicted Age (years)
Age (years)
b
Cell-type-specific bootstrapped pseudobulk aging clocks predictions (Velmeshev et al)
Oligodendrocytes
r = -0.025 (n.s.)
MAE = 19.88
Astrocytes
r = 0.359 (n.s.)
MAE = 20.13
Microglia
r = 0.528 (n.s.)
MAE = 21.34
OPCs
r = 0.693
MAE = 14.61
Inhibitory Neurons
r = 0.683
MAE = 23.66
Excitatory Neurons
r = 0.569 (n.s.)
MAE = 11.75
Predicted Age (years)
Age (years)
c
Performance of cell-type-specific single-cell aging clocks (Velmeshev et al)
Oligodendrocytes
Astrocytes
Microglia
OPCs
Excitatory Neurons
Inhibitory Neurons
Pearson r
MAE (years)
Single-cell
Simple pseudobulk
Bootstrapped pseudobulk
ns
d
Non-cell-type-specific aging clocks predictions (Velmeshev et al)
All-cells pseudobulk
r = 0.497 (n.s.)
MAE = 25.61
Glia-level pseudobulk
r = 0.304 (n.s.)
MAE = 23.35
Neuron-level pseudobulk
r = 0.664
MAE = 16.79
Predicted Age (years)
Age (years)
e
Performance of non-cell-type-specific aging clocks (Velmeshev et al)
All-cells pseudobulk
Glia-level pseudobulk
Neuron-level pseudobulk
Pearson r
MAE (years)
Simple
Bootstrapped 500
Bootstrapped 100
ns

Figure S6. Validation of aging clocks on Velmeshev et al. dataset[32]. Related to Figure 4. All plots show results from the validation of various aging clocks in Velmeshev et al dataset[32]. (a-b) Relationship between chronological age and predicted age in each cell, upon using (a) the cell-type-specific single-cell aging clock, and (b) the cell-type-specific bootstrapped-pseudobulk aging clock approaches. (c) Bar plots showing Pearson's correlation coefficients and mean absolute errors (MAE, represented by the intensity of blue colour in the bars) of each of the cell-type-specific approaches in each cell type. (d) Relationship between the chronological age and the predicted age based upon using the different non-cell-type-specific pseudobulk clock approaches. (e) Bar plots showing Pearson's correlation coefficients and mean absolute errors (MAE, represented by the intensity of green colour in the bars) of each of the non-cell-type-specific approaches. All correlation tests were performed using stats.pearsonr function of SciPy with significance based on a p-value < 0.05.

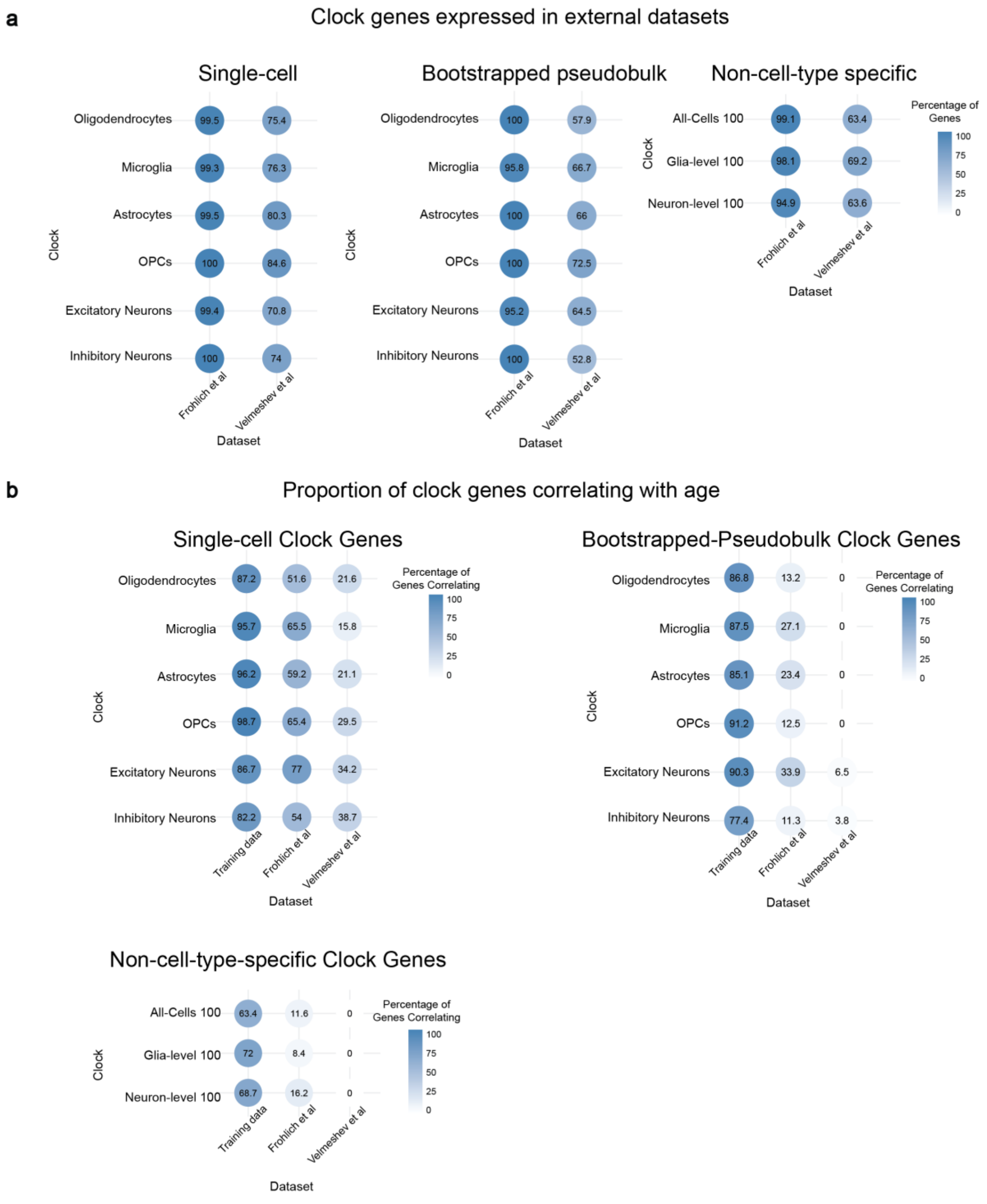

Figure S7. Proportion of clock feature genes expressed in the independent validation datasets. Related to Figure 4. (a) Plots showing the percentage of genes expressed in the external validation datasets, from the trained clock feature set. (b) Plots showing the proportion of clock genes, from all the trained clocks, showing a significant correlation with age, in the same direction as the average regression coefficient of the respective genes in the clock models.

Correlation was computed using Spearman's correlation test, followed by Benjamini-Hochberg correction for multiplicity. Adjusted p-value < 0.05 was considered significant.

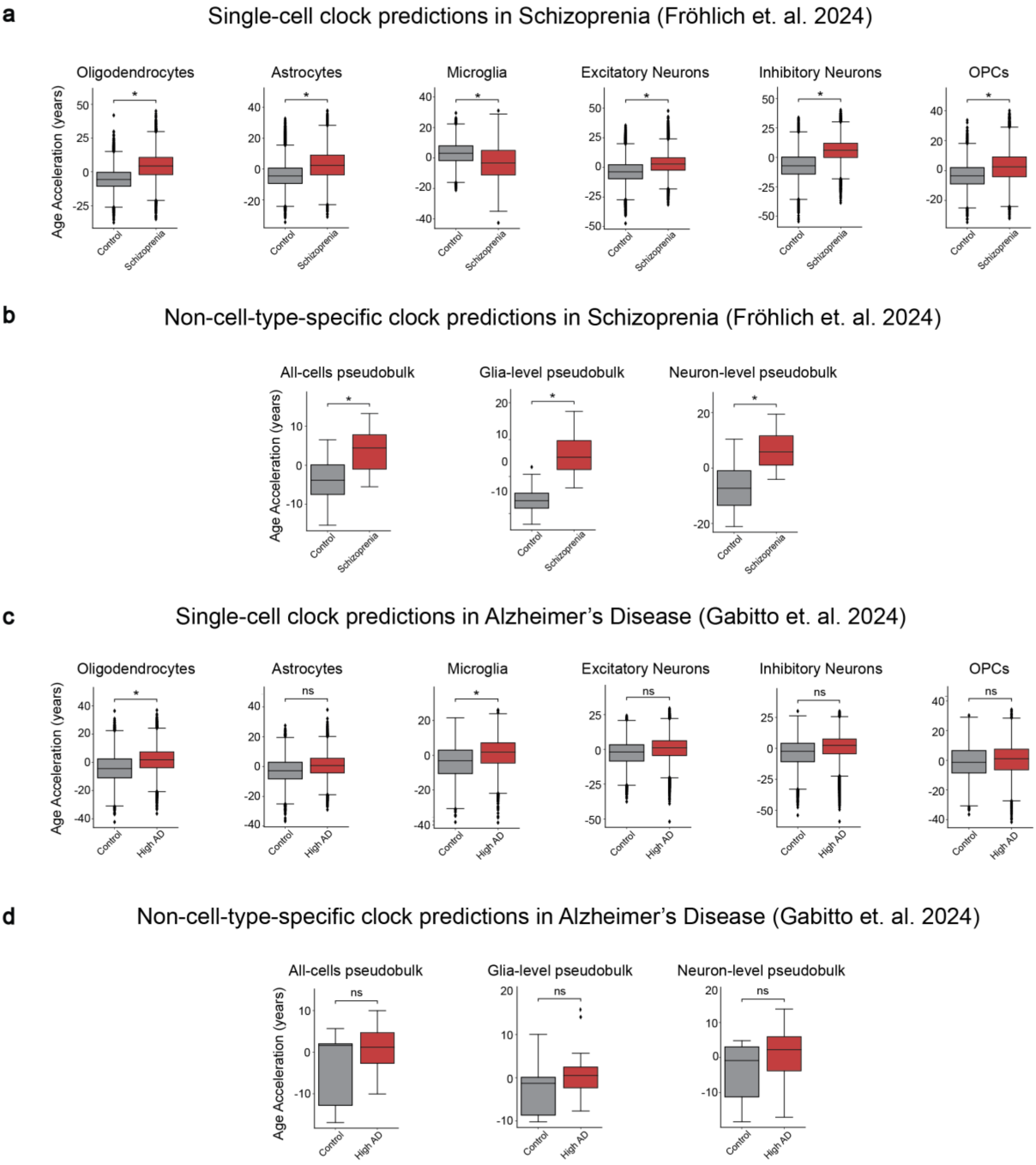

Figure S8. Prediction of age in age-related neurological disorders. Related to Figure 5. (a-d) The box plots show the age acceleration from the application of clocks in the single-nuclei RNA sequencing data of postmortem prefrontal cortex tissue from (a-b) Fröhlich et al.[18] and (c-d) Gabitto et al.[46]. In (a) and (c), the results were computed from single-cell clock predictions in Schizophrenia and High-AD (red), respectively, vs the controls (grey) in each of the major cell types. In (b) and (d), the results were computed from non-cell-type-specific clock predictions in Schizophrenia and High-AD (red), respectively, vs the controls (grey) across all the major cell types, glial cells or neuronal cells. $*p < 0.05$; Mixed Linear Model was used in (a) and (d), and Generalized Linear Model in (b) and (d) ('Application and evaluation of aging

clocks on neurological disorder datasets') to determine the significance of the difference between the age acceleration of control and disease samples.
